# The small acid-soluble proteins of *Clostridioides difficile* regulate sporulation in a SpoIVB2-dependent manner

**DOI:** 10.1101/2023.05.17.541253

**Published:** 2023-05-18

**Authors:** Hailee N. Nerber, Marko Baloh, Joseph A. Sorg

**Affiliations:** Department of Biology, Texas A&M University, College Station, TX 77845

## Abstract

*Clostridioides difficile* is a pathogen whose transmission relies on the formation of dormant endospores. Spores are highly resilient forms of bacteria that resist environmental and chemical insults. In recent work, we found that *C. difficile* SspA and SspB, two small acid-soluble proteins (SASPs), protect spores from UV damage and, interestingly, are necessary for the formation of mature spores. Here, we build upon this finding and show that *C. difficile sspA* and *sspB* are required for the formation of the spore cortex layer. Moreover, using an EMS mutagenesis selection strategy, we identified mutations that suppressed the defect in sporulation of *C. difficile* SASP mutants. Many of these strains contained mutations in *CDR20291_0714* (*spoIVB2*) revealing a connection between the SpoIVB2 protease and the SASPs in the sporulation pathway. This work builds upon the hypothesis that the small acid-soluble proteins can regulate gene expression.

## Introduction

*Clostridioides difficile* is a Gram-positive pathogen that causes approximately 220,000 cases of infection and nearly 13,000 deaths annually [1]. *C. difficile* vegetative cells produce toxins that disrupt the colonic epithelium, resulting in diarrhea and colonic inflammation [2, 3]. These toxin-producing vegetative cells are strictly anaerobic and cannot survive outside of a host for extended periods [4]. However, *C. difficile* produces endospores that are shed into the environment, can withstand oxygen and other environmental insults, and serve as the transmissive form of the organism [5–7].

Endospores are highly structured forms of bacteria. Residing in the spore core are the DNA, RNA, ribosomes, calcium dipicolinic acid (Ca-DPA), small acid-soluble proteins (SASPs), and other proteins necessary for the spore to outgrow into a vegetative cell [8–13]. Surrounding the core is a phospholipid membrane, cell wall, and a specialized cortex peptidoglycan layer. In the cortex, many of the N-acetylmuramic acid residues are converted into muramic-δ-lactam residues, which are recognized by the spore cortex lytic enzymes during germination [8, 14–16]. Outside of the cortex is a phospholipid outer membrane, a proteinaceous spore coat, and an exosporium [8, 17–21].

Generally, endospores are formed in response to nutrient deprivation. Upon initiation of sporulation, the vegetative cell asymmetrically divides into the larger mother cell and the smaller forespore compartments [22, 23]. The forespore becomes engulfed by the mother cell so that it can be matured into the dormant endospore. Once the endospore is fully formed, the mother cell lyses and releases the spore into the environment [24].

Like all known endospore-forming bacteria, the *C. difficile* sporulation program initiates upon phosphorylation of the sporulation master transcriptional activator, Spo0A [5, 25, 26]. After asymmetric division, each compartment begins a cascade of sigma factor activation [27, 28]. In the mother cell compartment, σ^E^ becomes activated and leads to σ^K^ expression. In the forespore compartment, σ^F^ is activated and leads to σ^G^ activation [8, 23]. A mutation in σ^F^ results in a strain that does not complete engulfment or form the cortex layer [28]. A mutation in σ^G^ results in a strain that forms a localized coat layer but does not fully complete engulfment or form the cortex layer. A mutation in σ^E^ results in a strain that is blocked at asymmetric septation. A mutation in σ^K^ results in a strain that fully engulfs the forespore and forms a correctly localized cortex layer, but no coat layer [28]. Thus, both engulfment and cortex assembly occur through σ^G^ regulated genes and coat production is dependent on σ^K^ genes.

The small acid-soluble proteins (SASPs) are very abundant in spores and have high sequence similarity across spore-forming species [29]. In many organisms, including *Bacillus subtilis* and *Clostridium perfringens,* the SASPs protect DNA against UV damage and damage from genotoxic chemicals [30–33]. In *B. subtilis*, the SASPs are considered non-specific DNA binding proteins that coat the DNA and change the conformation to a more rigid, intermediate, B to A form [29, 34–37]. This conformation leads to difficulty in forming UV-induced thymidine-dimers and, instead, promotes the formation of spore photoproducts; a repair mechanism is present in the spore to correct these lesions [38–40]. In *in vitro* transcription assays, addition of SASPs to DNA reduced transcription of some, but not all, genes, further illustrating their ability to bind DNA [37]. Moreover, the absence of transcription in mutant strains whose spores cannot degrade SASPs, suggest that SASPs could regulate gene expression [37, 41].

In prior work, we found that the *C. difficile* SASPs are important for spore UV resistance but do not strongly contribute to chemical resistances [42]. Surprisingly, a *C. difficile* Δ*sspA* Δ*sspB* double mutant strain could not complete spore formation, a phenotype not observed in other endospore-forming bacteria. This led us to hypothesize that the *C. difficile* SASPs are involved, somehow, in regulating sporulation. We hypothesize that SASPs have regions of high affinity on DNA where they bind to repress transcription of genes. As the concentration of SASPs increases, they nonspecifically coat the DNA to protect it. In the *C. difficile* Δ*sspA* Δ*sspB* strain, we hypothesize that sporulation is reduced due to altered gene expression of important sporulation genes.

Using a strategy that selected for the generation of mature spores from the sporulation deficient *C. difficile* Δ*sspA* Δ*sspB* strain, we identified mutations in *spoIVB2* that suppressed the mutant phenotype. SpoIVB2 is a protease that is uncharacterized in *C. difficile* and the *C. diffiicle* Δ*spoIVB2* mutant strain has a phenotype to similar the *C. difficile* Δ*sspA* Δ*sspB* strain. We hypothesize that the σ^G^-dependent expression of the *C. difficile* SASPs repress the σ^F^-dependent expression of *spoIVB2*, and that prolonged accumulation of SpoIVB2 in a *C. difficile* Δ*sspA* Δ*sspB* mutant halts sporulation by an unknown mechanism.

## Results

### C. difficile sspA and sspB regulate sporulation in the C. difficile CD630Δerm strain

**Figure 1.**
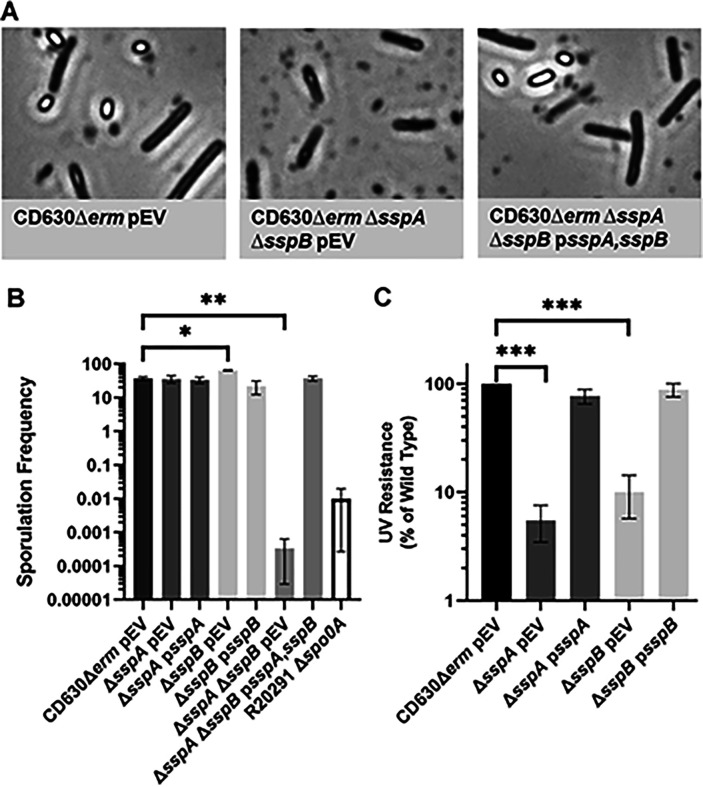
Impact of *C. difficile* CD630Δ*erm sspA* and *sspB* mutations on sporulation and UV resistance. A) Day 6 sporulating cultures were fixed in 4% formaldehyde and 2% glutaraldehyde in PBS and imaged on a Leica DM6B microscope. B) Strains were grown on 70:30 sporulation medium for 48 hours and the cultures then were treated with 30% ethanol and plated onto rich medium supplemented with TA to enumerate spores. An untreated culture was plated onto rich medium to enumerate vegetative cells. Sporulation frequency was calculated by [CFUs from spores / total cell CFUs (spores + vegetative cells)] *100. C) Spores were exposed to UV for 10 minutes with constant agitation. After treatment, they were serially diluted and plated onto rich medium supplemented with TA. The ratio of treated to untreated CFUs of the mutant strains was then compared to the ratio from WT. pEV indicates an empty plasmid within the strain. All data points represent the average of three independent experiments. Statistical analysis by one-way ANOVA with Šίdák’s multiple comparisons test. * P<0.01, ** P<0.001, *** P<0.0001.

In prior work, we discovered that *C. difficile* SspA and SspB were, individually, important for UV resistance [42]. Surprisingly, we found that the combinatorial deletion of the *sspA* and *sspB* genes, or a deletion in *sspB* and an *sspA*_G52V_ missense mutation (referred to as *C. difficile* ΔsspB* hereafter), in the *C. difficile* R20291 strain resulted in the drastic reduction of mature spore formation and, instead, resulted in phase gray spores [42]. To confirm that this phenotype was strain independent, we generated the single and double deletions of *sspA* and *sspB* in the *C. difficile* CD630Δ*erm* strain. Unsurprisingly, the CD630Δ*erm* Δ*sspA* Δ*sspB* double mutant also produced phase gray spores that were trapped within mother cells (Figure 1A). Though the single mutants did not affect sporulation frequency, the double mutant had a 5-log_10_ decrease in sporulation frequency. This defect could be restored to near wildtype levels by expression of *sspA* and *sspB*, *in trans*, from a plasmid (Figure 1B). When UV resistance was assessed, the *C. difficile* CD630Δ*erm* Δ*sspA* and the Δ*sspB* single mutant strains both had an approximate 1-log_10_ loss in viability after 10 minutes of UV exposure (Figure 1C). Though this is consistent with the findings we observed for the *C. difficile* R20291 Δ*sspB* mutant strain, the impact on viability for the *sspA* mutant was less in the *C. difficile* CD630Δ*erm* background than in *C. difficile* R20291 background. SspA and SspB appear to regulate sporulation in both the *C. difficile* R20291 and CD630Δ*erm* strains, hence, this is likely a conserved function in *C. difficile*.

### B. subtilis sspA complements UV and sporulation phenotypes of C. difficile R20291 mutants.

**Figure 2.**
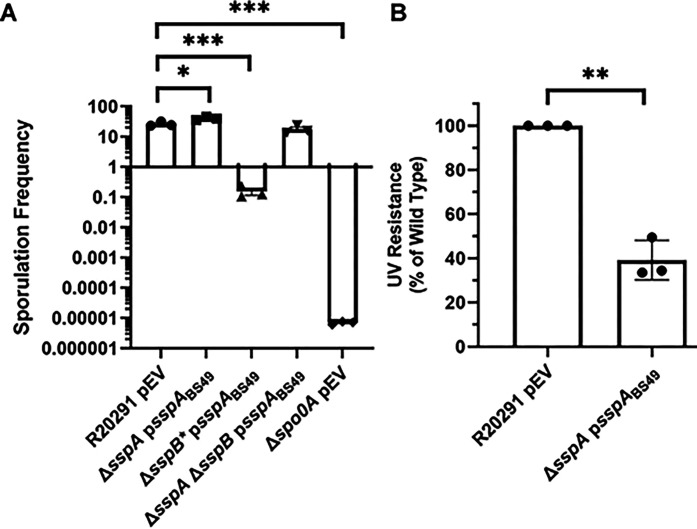
*B. subtilis sspA* complements *C. difficile* SASP mutant sporulation and UV defects. A) Sporulation frequency of the indicated strain was determined as described in Figure 1. B) Spores were exposed to UV for 10 minutes with constant agitation. After treatment, they were serially diluted and plated onto rich medium supplemented with TA. The ratio of treated to untreated CFUs of the mutant strains was then compared to the ratio from WT. pEV indicates an empty plasmid within the strain. All data points represent the average of three independent experiments. Statistical analysis by A) one-way ANOVA with Tukey’s multiple comparisons test and B) unpaired T-test. * P<0.05, ** P<0.001, *** P<0.0001.

Due to the high sequence similarity of SASPs, and their ability to cross-complement in other organisms, we assessed whether *sspA* from *B. subtilis* would complement the phenotypes observed in the *C. difficile* SASP mutants [29, 43]. The expression of *B. subtilis sspA* from the *C. difficile sspA* promoter in *C. difficile* ΔsspB* and *C. difficile* Δ*sspA* Δ*sspB* complemented the sporulation defect to varying degrees. In *C. difficile* ΔsspB*, expression of *B. subtilis sspA* increased sporulation by approximately 10-fold. In the *C. difficile* Δ*sspA* Δ*sspB* strain, expression of *B. subtilis sspA* increased sporulation by approximately 100-fold (Figure 2A).

Spores derived from a *C. difficile* Δ*sspA* mutant strain with a plasmid expressing *B. subtilis sspA*, under the *C. difficile sspA* native promoter, were exposed to UV light for 10 minutes and their viability assessed. *B. subtilis sspA* could partially restore UV resistance to the *C. difficile* Δ*sspA* mutant strain, although not to wild type levels (Figure 2B). These data show that *B. subtilis* and *C. difficile* SspA could function in similar ways due to the ability of *B. subtilis sspA* to complement phenotypes found in *C. difficile* SASP mutants.

### Visualizing the impact of SASP mutations on C. difficile spores

**Figure 3.**
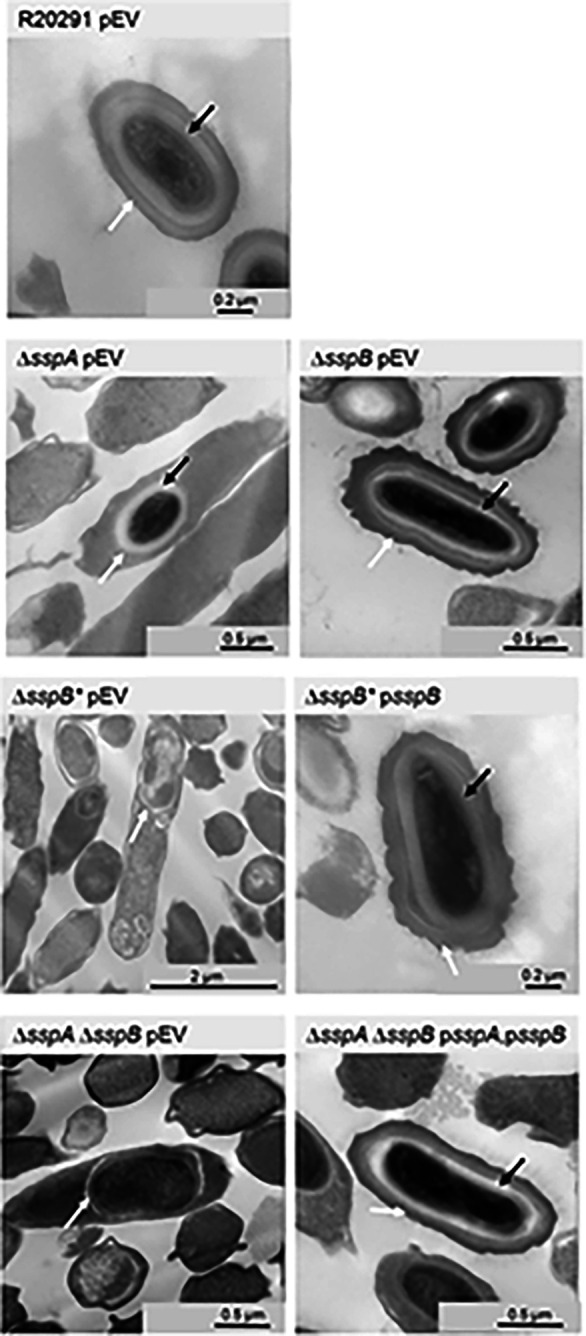
SASP mutants do not form the cortex layer. Day 6 sporulating cultures of wild type and mutant strains containing an empty vector (pEV) or the indicated plasmids were prepared for TEM. The coat layer is indicated with a white arrow, while the cortex layer is indicated with a black arrow.

To visualize the impact of the *C. difficile* Δ*sspA* Δ*sspB* deletions on spore structure, we used transmission electron microscopy (TEM). Strains generated in the *C. difficile* R20291 background were cultured for 6 days and then prepared for TEM. As expected, the *C. difficile* R20291 wild type strain generated fully formed and mature spores. The *C. difficile* Δ*sspA* and *C. difficile* Δ*sspB* single mutant strains also formed spores with the expected spore structures (*e.g.*, cortex and coat layers). However, *C. difficile* ΔsspB* and *C. difficile* Δ*sspA* Δ*sspB* strains generated spores that did not form cortex layers (Figure 3). Expression of the SASPs *in trans* under their native promoter regions complemented the mutant phenotypes by restoring the cortex layer and overall spore morphology.

### Isolating suppressor mutations of the SASP mutant phenotypes

To gain insight into how the SASPs are involved in spore formation, we used ethylmethane sulphonate (EMS) to introduce random mutations into the *C. difficile* genome, as we have previously done (Supplement Figure 1) [44, 45]. The *C. difficile* ΔsspB* or the *C. difficile* Δ*sspA* Δ*sspB* strains were treated with EMS, washed, and then incubated for 5 days to generate potential spores. Subsequently, the samples were heat-treated to kill vegetative cells and immature spores. After removing cellular debris, the cultures were plated on a medium supplemented with germinant and allowed to sporulate [46]. Subsequently, we isolated strains and confirmed that they generated spores. After confirmation, gDNA was extracted and sequenced to reveal the locations of potential mutations. From independent EMS mutageneses, we identified 4 suppressor strains generated from the *C. difficile* ΔsspB* strain and 10 from the *C. difficile* Δ*sspA* Δ*sspB* strain. Unsurprisingly, there were many mutations in each strain, but mutations that potentially contributed to suppression of the phenotype are listed in Table 1 (a full list of mutations can be found in Table S3). As expected, due to the strong selection for spore dormancy, 2 out of 4 of the isolates from *C. difficile* ΔsspB* had a reversion mutation in *sspA*. We identified mutations in different RNA polymerase subunits in 7 of 14 strains. These mutations could potentially affect transcription rates of various genes. Mutations within the *sigG* and *spoVT* genes were also present in some strains. *sigG* and *spoVT* mutants have a similar phenotype to the *C. difficile* Δ*sspA* Δ*sspB* strain [27, 28, 47, 48]. Interestingly, 7 out of 14 isolates (from several mutageneses) contained mutations in *CDR20291_0714*. Among these strains, we observed one strain with an A20T missense mutation and six with a synonymous mutation (F37F). The *C. difficile* CD630Δ*erm* genome encodes a gene homologous to *CDR20291_0714* and is annotated as *spoIVB2*. SpoIVB2 is a paralog of the SpoIVB protease, and we refer to CDR20291_0714 as SpoIVB2 from here on.

**Figure 4.**
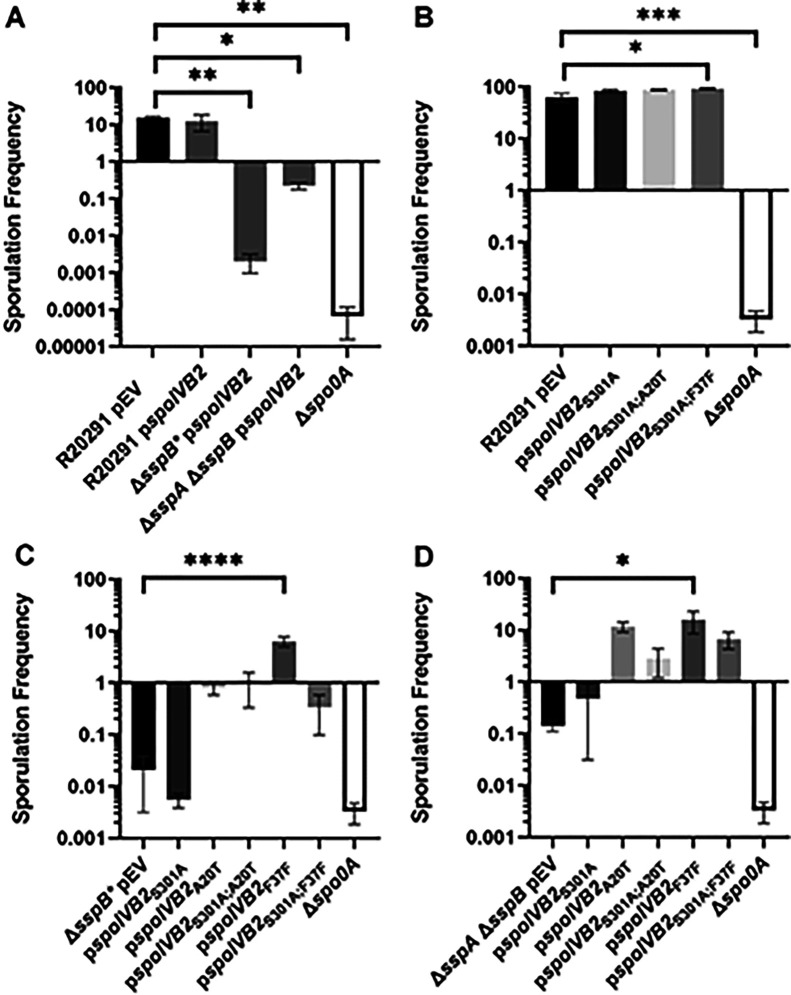
Mutations in *spoIVB2* suppress the *C. difficile sspA sspB* mutant phenotype. Sporulation frequency of the indicated strain was determined as described in Figure 1. B) Sporulation of *C. difficile* R20291 with the indicated *spoIVB2* allele expressed from a plasmid. C) Sporulation assay of *C. difficile* R20291 ΔsspB* with the indicated *spoIVB2* allele expressed from a plasmid. D) Sporulation assay of R20291 Δ*sspA* Δ*sspB* with the indicated *spoIVB2* allele expressed from a plasmid. pEV indicates an empty plasmid within the strain. All data points represent the average from three independent experiments. Statistical analysis by ANOVA with Dunnett’s multiple comparisons test. * P<0.05, ** P<0.01, *** P<0.001, **** P<0.0001.

We first tested if *in trans* expression of the identified *spoIVB2* alleles could restore sporulation to the SASP mutant. Unfortunately, due to changes in peptone availability (supply chain problems), the sporulation frequency of the strains varies between Figure 4A and Figures 4B–D. Thus, inter-panel comparisons should not be made; the medium used in Figure 4A resulted in a sporulation frequency of approximately 10%, while the medium used in Figure 4B–D yielded an approximate 60% sporulation frequency for the wild type *C. difficile* R20291 strain. We also tested if catalytic activity impacted restoration. The catalytic site was identified by aligning *C. difficile* SpoIVB / SpoIVB2 to *B. subtilis* SpoIVB. The three catalytic residues found in *B. subtilis* are conserved in both SpoIVB and SpoIVB2 of *C. difficile* and we have used *spoIVB2*_S301A_ as a catalytically dead mutant [49]*.* When wild type *spoIVB2* was expressed in *C. difficile* R20291, *C. difficile* ΔsspB*, or the *C. difficile* Δ*sspA* Δ*sspB* strains, the sporulation frequency did not change from their respective phenotype (Figure 4A). Moreover, in the wildtype *C. difficile* R20291 strain, the sporulation frequency was not impacted when the *spoIVB2*_A20T_ or *spoIVB2*_F37F_ alleles were combined with S301A (Figure 4B).

When the *spoIVB2* alleles were introduced into the *C. difficile* ΔsspB* strain, the *spoIVB2*_S301A_ allele did not restore sporulation frequency, but the *spoIVB2*_A20T_ and *spoIVB2*_F37F_ alleles increased sporulation by approximately 2 and 2.5 log_10_, respectively. Whenever these alleles were combined with the *spoIVB2*_S301A_ allele, sporulation was still restored, albeit to a lesser extent (Figure 4C). These results were similar to when the *spoIVB2* alleles were expressed in the *C. difficile* Δ*sspA* Δ*sspB* strain [the expression of *spoIVB2*_A20T_ and *spoIVB2*_F37F_ resulted in an approximate 2 log_10_ increase in sporulation frequency (Figure 4D)]. The catalytically dead allele in combination with the identified alleles from EMS was again able to restore sporulation frequency but to a lower, non-significant, level. These results suggest that *C. difficile* SpoIVB2 has some role outside of its catalytic activity. However, because wild type SpoIVB2 is present within these strains, the requirement for SpoIVB2 catalytic activity in these plasmids may be lessened.

From these data, we hypothesized that SspA and SspB are repressing the expression of *spoIVB2*. We hypothesize that in the *C. difficile* Δ*sspA* Δ*sspB* and the *C. difficile* ΔsspB* mutants there is an overabundance of SpoIVB2, which leads to early processing of or binding to a target that is essential for sporulation. Therefore, expression of *spoIVB2* (Figure 4A) does not restore the sporulation deficient phenotype. The suppressor strains potentially reduce the amount of SpoIVB2 present. To test this, we expressed wild type *spoIVB2* (from a plasmid) in the suppressor strains that have *spoIVB2*_A20T_ or *spoIVB2*_F37F_ and quantified spore formation. We found the general trend is for sporulation frequency to decrease when wild type *spoIVB2* is expressed from the plasmid, but these differences did not meet statistical significance (Supplementary Figure 2).

### The C. difficile spoIVB2 mutant is phenotypically similar to the C. difficile ΔsspA ΔsspB strain

**Figure 5.**
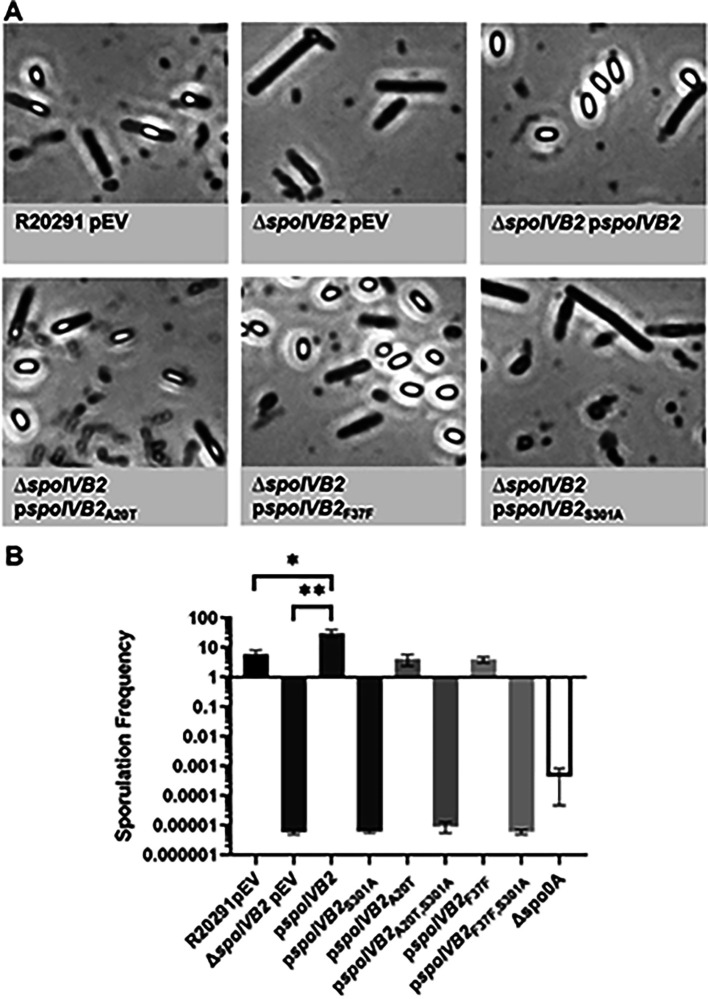
*C. difficile* Δ*spoIVB2* has a sporulation defect. A) Day 6 cultures were fixed in 4% formaldehyde and 2% glutaraldehyde in PBS and imaged on a Leica DM6B microscope. B) Sporulation frequency of the indicated strain was determined as described in Figure 1. pEV indicates an empty plasmid within the strain. All data points represent the average from three independent experiments. Statistical analysis by one-way ANOVA with Šίdák’s multiple comparisons test. * P<0.001, ** P<0.0001.

To further evaluate the role of SpoIVB2 during sporulation, we generated a deletion of *spoIVB2* in the *C. difficile* R20291 strain. The *C. difficile* Δ*spoIVB2* strain generated phase gray spores, similar to our observations for the *C. difficile* Δ*sspA* Δ*sspB* strain (Figure 5A). This phenotype could be complemented by expression of *spoIVB2*_WT_, *spoIVB2*_A20T_, or *spoIVB2*_F37F_ alleles from a plasmid. However, restoration did not occur when the catalytically dead *spoIVB2*_S301A_ was expressed, highlighting the importance of catalytic activity in the function of SpoIVB2 (Figure 5A). The sporulation frequency of the *C. difficile* Δ*spoIVB2* strain was 6-log_10_ lower than wild type. The *C. difficile* Δ*spoIVB2* mutant supplemented with a plasmid expressing *spoIVB2* wild type, A20T or F37F alleles restored the sporulation frequency to wild type levels (Figure 5B). However, whenever the S301A allele is present or in combination with the A20T or F37F alleles, sporulation is not restored, highlighting the necessity for catalytic activity in the function of SpoIVB2 (Figure 5B).

**Figure 6.**
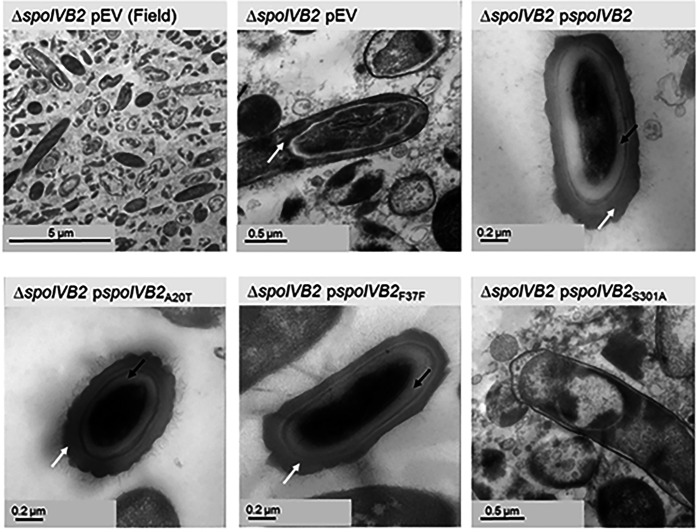
SpoIVB2 is required for cortex synthesis. Day 6 sporulating cultures were prepared for TEM. A field of view image is shown for the *C. difficile* Δ*spoIVB2* mutant while the remainder of the images are zoomed into the sporulating cell / spore. The coat layer is indicated with a white arrow, while the cortex layer is indicated with a black arrow. pEV indicates an empty plasmid within the strain.

Analysis of the *C. difficile* Δ*spoIVB2* strain by TEM revealed many problems with the sporulating cells (Figure 6). As seen in the field of view image, it was difficult to locate whole cells for imaging. When a sporulating cell was found, there were structural issues within the forespore. The cortex was missing and, with the lack of its constraint around the core, allowed for expansion of the core contents. Whenever *spoIVB2*_WT_, *spoIVB2*_A20T_, or *spoIVB2*_F37F_ were expressed from a plasmid, the structural appearance of the spore was restored to wild type. However, when *spoIVB2*_S301A_ was expressed, it remained difficult to locate any sporulating cells.

### Testing the impact of the suppressor alleles on SpoIVB2 expression

To understand how the SASPs influence *spoIVB2* and / or other gene transcripts, RNA was extracted 11 hours post plating on sporulation medium and qRT-PCR was performed. Overall, at this time point, there were few differences in transcript levels. The *spoIVB2*_A20T_ isolate (HNN19) was variable between extractions despite adding more trial, potentially due to other mutations from the EMS treatment. Though *sspA* or *sspB* transcripts levels are largely unchanged, there’s a slight increase in transcript levels for *spoIVA*; a protein that is involved in spore coat localization (Supplementary Figure 3A–C). Transcripts for *sleC*, *pdaA,* and *spoVT* remained similar to wild type levels (Supplementary Figure 3D–F). *spoIVB* transcript levels did not have a concise trend while, for *spoIVB2*, the general trend is towards slightly reduced transcripts in the mutant strains with a larger fold change in the EMS identified alleles (Supplementary Figure 4A–B). *spoIIP* transcripts are slightly elevated in the mutant strains, except for the EMS isolates (Supplementary Figure 4C). For the DPA synthesis and packaging protein transcripts (*dpaA*, *spoVAC*, *spoVAD*, and *spoVAE*), there were minimal differences for the mutant strains besides a slight increase in *spoVAC* (Supplementary Figure 5A–D).

**Figure 7.**
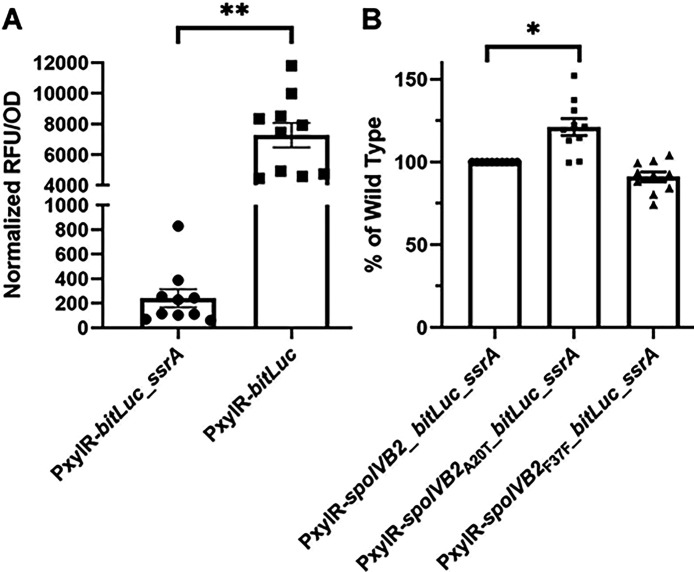
Luciferase assays show minimal differences between *spoIVB2* protein levels. Steady-state protein levels of the indicated strain were determined using alleles with an engineered *ssrA* tag (as described in the materials and methods). A) The RFU/OD_600_ of uninduced culture was subtracted from the RFU/OD_600_ of the induced culture. B) The RFU of uninduced culture was divided by the OD_600_ and then subtracted from the ratio of the induced culture. The *spoIVB2* alleles identified in EMS were then compared to the wild type *spoIVB2* construct value. All data represents the average of ten independent experiments. Statistical analysis by A) unpaired t-test or B) ANOVA with Šίdák’s multiple comparison test. * P<0.001, ** P<0.0001.

We hypothesized that the *spoIVB2*_F37F_ allele restores sporulation through translational differences, rather than transcriptional. To test this hypothesis, we designed a luciferase-based assay [50–52] to test the differences in translation between *spoIVB2* alleles. To ensure that we observe steady-state levels of the luciferase reporter, we engineered a *ssrA* tag to the 3’ end of the gene. This tag targets the protein for degradation by the ClpP protease [53]. As a control, the *bitLuc* gene with and without the *ssrA* tag was put under control of a xylose-inducible promoter. After expression, the construct containing the *ssrA* tag had significantly lower normalized RFU / OD than the construct without the tag (Figure 7A). This shows that the *ssrA* tag reduces luciferase abundance. We coupled a xylose driven promoter to the *bitLuc* gene and 60 codons of either wild type *spoIVB2, spoIVB2*_A20T_, or *spoIVB2*_F37F_ and tagged the construct for degradation with a *ssrA* tag. After 30 minutes of expression, the *spoIVB2*_A20T_ construct had higher levels of luminescence compared to wild type. Even though the F37F construct had lower RFU than the wild type construct in 8 out of 10 trials, this difference was minimal (with 7 out of 10 trials having greater than 5% difference) and not statistically significant (Figure 7B).

**Figure 8.**
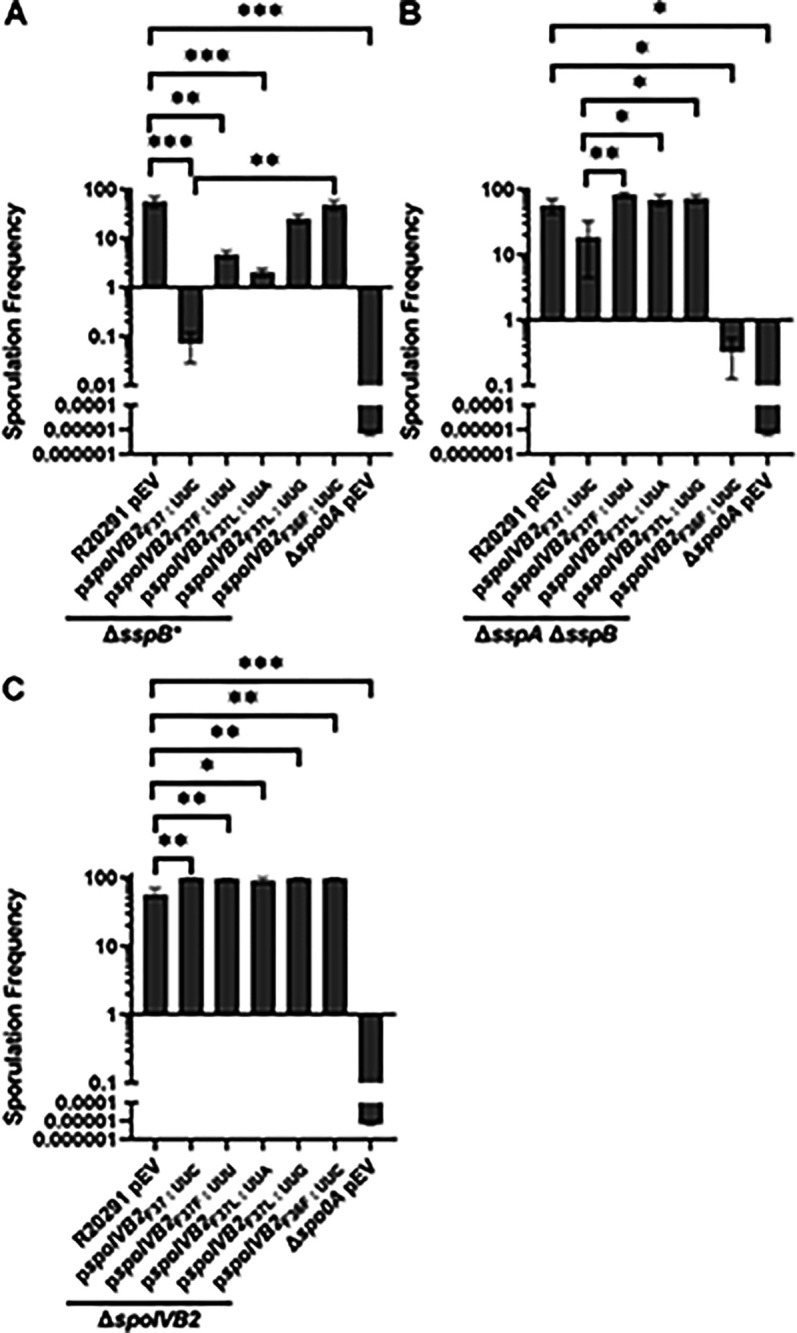
Manipulation of *spoIVB2*_F36 / F37_ restores sporulation. Sporulation frequency of the indicated strain was determined as described in Figure 1.. The plasmids expressing the *spoIVB2*_F37_ alleles and the *spoIVB2*_F36F_ allele were assessed in the strains A) *C. difficile* ΔsspB*, B) *C. difficile* Δ*sspA* Δ*sspB*, C) *C. difficile* Δ*spoIVB2.* pEV indicates an empty plasmid within the strain. All data represents the average of three independent experiments. Statistical analysis by ANOVA with Šίdák’s multiple comparison test. * P<0.05, ** P<0.01, *** P<0.001.

Because the translational differences between *spoIVB2* alleles was minimal, we next manipulated the F37 codon to see if other changes would allow for sporulation to be restored in the mutant strains. We also changed the F36 codon from UUU to UUC (generating an F36F silent mutation and the opposite codon change that occurred in the F37F allele). These constructs were expressed from a plasmid under the *spoIVB2* native promoter region, and the sporulation frequency was assessed. When wild type *spoIVB2* (UUC codon) is expressed in the *C. difficile* ΔsspB* strain, sporulation was not restored to wild type levels (Figure 8A). However, sporulation was partially restored with the *spoIVB2*_F37F_ (UUU codon) and the *spoIVB2*_F37L_ (UUA codon) alleles. Furthermore, the sporulation frequency was similar to wild type levels when *spoIVB2*_F37L_ (UUG codon) and *spoIVB2*_F36F_ (UUC codon) were expressed (Figure 8A). This suggests that manipulation of this region of *spoIVB2* is sufficient to restore sporulation in an otherwise sporulation deficient strain.

Expression of these plasmids in the *C. difficile* Δ*sspA* Δ*sspB* double mutant strains showed variation from the previously assessed strain. First, expression of the wild type *spoIVB2* allele resulted in an increase in sporulation frequency with levels that were similar to wild type (Figure 8B). This differs from the sporulation frequency determined in Figure 4A. The reason for this could be the differences in peptone used between studies, which altered the amounts of spores that could be produced. However, expression of the *spoIVB2*_F37F_ (UUU codon) or the *spoIVB2*_F37L_ (UUA or UUG codons) restored the sporulation frequency to a higher level than the wild type *spoIVB2* allele. Interestingly, in the *C. difficile* Δ*sspA* Δ*sspB* strain, *spoIVB2*_F36F_ did not complement the sporulation phenotype as it did in the *C. difficile* ΔsspB* strain (Figure 8B).

Finally, expression of any of the *spoIVB2* alleles restores sporulation in the *C. difficile* Δ*spoIVB2* mutant strain (Figure 8C). These data suggest that altering the F37 codon in either of the sporulation deficient strains can restore sporulation.

## Discussion

The formation of endospores in *C. difficile* is vital for transmission of disease and the mechanisms involving spore formation are complex [8]. In prior work, we determined that the *C.* Δ*sspA* Δ*sspB* strain was halted during sporulation suggesting that the *C. difficile* SASPs are important for regulating late-stage sporulation, somehow [42]. Here, we built upon our findings by further exploring the SASP mutant strain using TEM and a selection strategy to identify potential suppressor mutants.

Oddly, during the course of the prior work, we identified a mutation in the *C. difficile sspA* gene during the generation of the *sspB* mutant using CRISPR-Cas9 editing. This strain, *C. difficile* Δ*sspB*; *sspA*_G52V_ (*C. difficile* ΔsspB*), had a phenotype similar to the *C. difficile* Δ*sspA* Δ*sspB* strain. This phenotype was likely due to the missense mutation within a conserved glycine residue. Prior work in *B. subtilis* found that SspC^G52A^ was unable to bind DNA [37, 54]. Oddly, we have since observed a similar off-target effect in the *sspB* gene when targeting *sspA* using CRISPR-Cas9 mutagenesis. During the process of targeting *sspA* in a *C. difficile* Δ*gpr* strain, an *sspB*_E64stp_ allele was also observed upon confirmation of the mutant’s DNA sequence. The two genes are not located in close proximity nor do the constructs for deletion encode this sequence. We hypothesize that there may be some selective pressure to mutate *sspA* and *sspB* within a deletion strain.

With further evaluation of SASP mutant strains by TEM, we found that the *C. difficile* Δ*sspA* Δ*sspB* strain produces forespores that are blocked after the engulfment step, and do not contain cortex. Cortex is synthesized by the σ^G^-controlled PdaA, GerS, and CwlD proteins. These proteins modify the peptidoglycan to generate muramic-δ-lactam residues [55–57]. The cortex provides a physical constraint around the spore core, maintaining size and preventing water from hydrating the Ca-DPA-rich core [8, 58]. In the absence of cortex, it is likely that some contents in the spore core leak out. This likely explains our previous findings that the few spores that could be purified from the *C. difficile* Δ*sspA* Δ*sspB* and the *C. difficile* ΔsspB* strains contained little CaDPA [42]. Because our qRT-PCR data showed that *dpaA* and *spoVAC/D/E* transcript levels are similar to wild type levels in these mutant strains, it is likely that DPA is being synthesized, and likely transported into the spore, but cannot be concentrated into the core without a mature cortex layer.

An EMS mutagenesis strategy to find suppressors of the defect in sporulation of the *C. difficile* Δ*sspA* Δ*sspB* strain identified mutations in *spoIVB2*. SpoIVB2 is homologous to *B. subtillis* SpoIVB. Though *B. subtilis* contains the same spore layers and sigma factors that regulate sporulation in *C. difficile*, the process is more complex. Sporulation is under control of a defined phosphorelay pathway to phosphorylate Spo0A [59]. Activated Spo0A~P leads to the production of Pro-σ^E^ in the mother cell and σ^F^ in the forespore. The sigma factors are activated through cross-talk between the forespore and mother cell compartments [22, 28]. σ^F^ activation leads to activation of σ^E^, σ^E^ activation leads to σ^G^ activation, σ^G^ activation to Pro-σ^K^ activation. The SpoIVB protease is produced under σ^G^ control. Located in the *B. subtilis* outer forespore membrane are the SpoIVFB, SpoIVFA, and BofA proteins [60]. BofA is an inhibitor of the SpoIVFB protease, and SpoIVFA keeps the proteins localized in the membrane. SpoIVB processes SpoIVFA, thereby relieving BofA inhibition of SpoIVFB. Activated SpoIVFB cleaves the pro-peptide from σ^K^, resulting in σ^K^ activation [60].

In addition to its role in σ^K^ activation, SpoIVB has other functions in *B. subtilis, e.g.* cleavage of SpoIIQ [61]. SpoIIQ is required for σ^G^ synthesis and contributes to the formation of a feeding tube between the mother cell and the forespore compartments. SpoIVB cleaves SpoIIQ upon completion of engulfment, however, this cleavage is not necessary for spore formation or any later-stage gene expression [61, 62]. A *spoIVB* null mutant blocks the formation of fully formed, heat resistant spores [63]. Spores derived from this strain form the forespore but lack the germ cell wall layer and did not generate mature spores. Interestingly, this phenotype was independent of SpoIVB’s role in the activation of σ^K^ [63]. An alternative role for SpoIVB may be in germ cell wall biosynthesis or as a DNA binding regulatory protein.

Even though the *C. difficile* sporulation program does not contain the cross-talk sigma factor activation or homologs to *bofA*, *spoIVFA*, or *spoIVFB*, it does contain the paralogs SpoIVB and SpoIVB2 [28]. SpoIVB2 is σ^F^-regulated while SpoIVB is σ^G^-regulated [47]. *C. difficile* SpoIVB and SpoIVB2 contain 31% identity to each other and have 36% and 37% identity to *B. subtilis* SpoIVB, respectively [47].

In our sporulation assays, *spoIVB2*_A20T_ and *spoIVB2*_F37F_ can rescue the mutant phenotype and form mature, dormant spores. While the *spoIVB2*_A20T_ allele might rescue the strain through changes in SpoIVB2 activity, we hypothesize that the F37F allele suppresses the phenotype through translational changes. Interestingly, in the identified *spoIVB2*_F37F_ strain, the wildtype UUC codon is used in 5.9 / 1000 codons but the UUU codon in the suppressor strain is used 37.4 / 1000 codons [64]. Also, out of the 18 phenylalanine residues found in the SpoIVB2 protein, only F37 uses the UUC codon. Even though the codon changes to one that is used more frequently, this data is based on codon usage across the whole *C. difficile* genome and not just spore specific genes.

**Figure 9.**
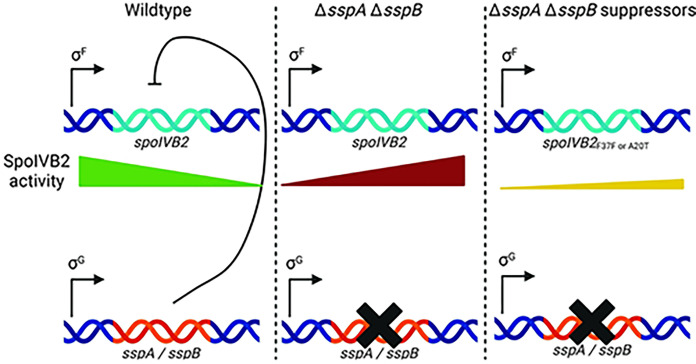
Model for *spoIVB2* regulation by the *C. difficile* SASPs. In a working model for how *C. difficile* SASPs regulate sporulation, we hypothesize that upon σ^G^ activation in the forespore of wildtype *C. difficile* cells, SspA and SspB become expressed, and they bind to *spoIVB2* to prevent prolonged accumulation of SpoIVB2. In the *C. difficile* Δ*sspA* Δ*sspB* or in the *C. difficile* ΔsspB^*^ strains, the SASPs do not accumulate and SpoIVB2 retains prolonged activity in the forespore compartment. In the *C. difficile* Δ*sspA* Δ*sspB* or in the *C. difficile* ΔsspB^*^ suppressor strains, the SpoIVB2^A20T^ or the *spoIVB2*_*F37F*_ alleles lead to lower amounts of SpoIVB2 activity (either through changes in protease activity or due to translational changes, respectively) later in sporulation. Created with BioRender.com.

In our working model for how the *C. difficile* SASPs influence *spoIVB2*, *w*e hypothesize that SASP binding could repress genes either by occluding promoter regions or by a roadblock mechanism. Because *spoIVB2* is under σ^F^-control, σ^G^-produced SASPS could block *spoIVB2* expression (Figure 9). In the absence of *C. difficile sspA* and *sspB*, SpoIVB2 activity continues into later stages of sporulation (Figure 9). Since the SASPs are not present in the suppressor strains, we wondered if the *spoIVB2* alleles identified altered translation rates and, thus, reduced SpoIVB2 activity (Figure 9). Though our BitLuc data did not show a change in RFU for translation of the F37F allele compared to wild type, it is possible that only a small change in translation is needed to restore the phenotype. When the wobble position of *spoIVB2*_F37_ was manipulated, sporulation was restored in both, the *C. difficile* Δ*sspA* Δ*sspB* mutant and the *C. difficile* ΔsspB* mutant strains (even though the F37F allele was only identified in the former strain). Also of note, it is likely that the mutation to the UUU codon was the only identified change after EMS treatment, instead of the UUA or UUG codons, due to the nature of EMS mutagenesis which results in transition mutations. We analyzed transcript variation among strains for various genes, including *spoIVB2.* In this data, the transcripts for *spoIVB2* in representative EMS strains, for both the *spoIVB2*_A20T_ and *spoIVB2*_F37F_ alleles, trended toward being downregulated, though this difference was only ~4 fold. Unfortunately, *C. difficile* sporulation is asynchronous and samples from any time point contain cells in every stage of sporulation. This could explain why the fold changes are small and variable across all strains and all transcripts analyzed. Therefore, it is difficult to draw definitive conclusions from the qRT-PCR data.

Separate from how the *spoIVB2* alleles restore sporulation to the SASP mutant strains, what is the function of SpoIVB2 during sporulation? While it is possible that the *C. difficile* SpoIVB and SpoIVB2 proteins retain a function in SpoIIQ cleavage, in *C. difficile,* SpoIIQ does not appear to be cleaved during *C. difficile* sporulation [65]. However, unlike in *B. subtilis*, *C. difficile* SpoIIP has a cleaved form that is only present in cells that have completed engulfment [65]. SpoIIP is an amidase and endopeptidase that works in concert with SpoIID to restructure peptidoglycan during forespore engulfment. In a *C. difficile* Δ*spoIIP* strain, the leading edge of engulfment does not progress, so engulfment does not occur [65]. Once engulfment is complete, it could be possible that SpoIIP needs to be cleaved before the following stages of sporulation can continue. As our SASP mutants appear to complete engulfment but arrest at cortex formation, SpoIIP may be a potential target for SpoIVB2.

Another strong possibility is that SpoIVB2 is necessary as a regulatory component for another gene(s), potentially for proteins involved in cortex formation. Though our data demonstrate that catalytic activity is required to complement a *C. difficile* Δ*spoIVB2* mutant strain, the *spoIVB2*_A20T_ and *spoIVB2*_F37F_ alleles did not require catalytic activity to partially restore the defect in sporulation when expressed in SASP mutants. This furthers the idea that the *spoIVB2* alleles can restore sporulation through a mechanism that is separate from its protease activity, such as a scaffolding or regulatory function [63].

To better understand if the SASPs function similarly among other organisms, we also tested the ability of the *B. subtilis sspA* gene to complement sporulation and UV phenotypes in *C. difficile* mutants. When expressed under the *C. difficile sspA* native promoter region, *B. subtilis sspA* can partially restore sporulation and UV resistance. In prior work from the Setlow lab [66], the authors suggest that SASPs may affect forespore transcription, likely by physically blocking RNA polymerase. Furthermore, *in vitro* transcription assays in *B. subtilis* show that less *in vitro* transcription occurs when SASPs are incubated with DNA. However, transcription occurs in the absence of SASPs or in the presence of a SASP variant with poor DNA binding ability [37]. These data indicate that the SASPs could be regulating sporulation in the forespore of both *C. difficile* and *B. subtilis*, suggesting that the different phenotypes observed between the Δ*sspA* Δ*sspB* double mutants in the two organisms lie in the differences between the mechanism of compartmental signaling during sporulation. This leads to further questions about whether the genes / regions of DNA that are influenced by the SASPs and how the SASPs may potentiate these affects differs between the two organisms.

Overall, this study gives insight into the sporulation process and regulation in *C. difficile.* It is likely that the SASPs have binding “hotspots” where in low concentrations they preferentially bind to influence transcription. Although the qRT-PCR data did not show many transcriptional changes, we hypothesize that the SASPs are influencing transcription of target genes. It is possible that the time of extraction was not ideal for capturing transcriptional changes, the change is small enough that the variability in data due to sporulation being asynchronous could be enough to hide the affects, or, though unlikely based on our data, the SASPs have different targets than those tested. This study also highlights the importance of *C. difficile* SpoIVB2 during sporulation even though its exact role is still unknown. Further work needs to be completed to understand the influence of SpoIVB2 during sporulation and to determine other potential targets for the SASPs.

## Materials and Methods

### Bacterial growth conditions:

*C. difficile* strains were grown in a Coy anaerobic chamber at ~4% H_2_, 5% CO_2_, and balanced N_2_ at 37 °C [67]. Strains were grown in / on brain heart infusion (BHI), BHI supplemented with 5 g / L yeast extract (BHIS), 70:30 (70% BHIS, 30% SMC) or tryptone yeast (TY) medium. 0.1% L-cysteine was added to BHI and BHIS while 0.1% thioglycolate was added to TY. Media was supplemented with thiamphenicol (10 μg / mL), taurocholate [TA] (0.1%), cycloserine (250 μg / mL), kanamycin (50 μg / mL), lincomycin (20 μg / mL), rifampicin (20 μg / mL), ethylmethane sulfonate [EMS] (1%), or D-xylose (0.5% or 1%) where indicated. *E. coli* strains were grown on LB at 37 °C and supplemented with chloramphenicol (20 μg / mL) or ampicillin (100 μg / mL). *B. subtilis* BS49 was grown on LB agar plates or in BHIS broth at 37 °C and supplemented with 2.5 μg / mL chloramphenicol or 5 μg / mL tetracycline.

### *Plasmid construction:* All cloning was performed in *E. coli* DH5α.

For construction of the *C. difficile* CD630Δ*erm sspA-*targeting CRISPR vector, pHN120, 500 bp of upstream homology was amplified from CD630Δ*erm* genomic DNA with primers 5’sspA_MTL and 3’sspA_UP while the downstream homology arms were amplified with 5’sspA_down and 3’ sspA_xylR. These were inserted into pKM197 at the *Not*I and *Xho*I sites using Gibson assembly [68]. The gRNA gBlock (Integrated DNA Technologies, Coralville, IA) CRISPR_sspA_165 was inserted at the *Kpn*I and *Mlu*I sites. pHN120 was then used as the base plasmid to change the *Tn916* oriT for the *traJ* oriT at the *Apa*I sites, resulting in pHN131. *traJ* was amplified from pMTL84151 with primers 5’traJ and 3’traJ. The gRNA was then replaced with gBlock CRISPR_sspA_135 at the *Kpn*I and *Mlu*I restriction sites, generating pHN138, which was used to make the deletion.

For generating the *C. difficile* CD630Δ*erm sspB* targeting CRISPR vector, pHN121, the upstream homology was amplified from CD630Δ*erm* genomic DNA with 5’ sspB UP and 3’ sspB UP and the downstream homology with 5’ sspB DN and 3’ sspB_xylR. These homology arms were inserted into pKM197 at *Not*I and *Xho*I restriction sites using Gibson assembly [68]. The gRNA gBlock CRISPR_sspB_144 was also inserted into the *Kpn*I and *Mlu*I sites. The oriT was changed from *Tn916* to *traJ* by amplifying *traJ* from pMTL84151 with 5’traJ and 3’traJ and inserting in the *apa*I sites to generate pHN132.

For generating the *C. difficile* R20291 *spoIVB2* targeted CRISPR plasmid, the upstream homology arm was amplified from R20291 with primers 5’ CDR20291_0714 UP and 3’ CDR20291_0714 UP while the downstream was amplified with 5’ CDR20291_0714 DN and 3’ CDR20291_0714 DN. These were inserted into pKM197 at the *Not*I and *Xho*I sites using Gibson assembly [68]. The gRNA was amplified from pKM197 using primers CDR20291_0714 gRNA 3 and 3’ gRNA_change. This fragment was inserted into the *Kpn*I and *Mlu*I sites using Gibson assembly, generating pHN157 [68].

Plasmid pHN149 was generated by amplifying the *traJ* oriT from pMTLYN4 with primers 5’ tn916.traJ and 3’traJ and the *Tn916* oriT from pJS116 with 5’Tn916ori_gibson and 3’ tn916.traJ. These were inserted into the A*pa*I site of pMTL84151.

For the generation of pHN122, pHN123, and pHN127, the *spoIVB2* gene and promoter regions were amplified from HNN37, HNN19, and R20291, respectively, using 5’ CDR20291_0714 and 3’ CDR20291_0714. These fragments were inserted into pJS116 (for pHN122 and pHN123) or pHN149 (for pHN127) at the *Not*I and *Xho*I sites using Gibson assembly [68].

For pHN145, pHN146, and pHN147, the first segment of DNA was amplified from R20291, pHN122, or pHN123, respectively, using 5’ CDR20291_0714 and 3’ 0714_S301A. The second segment of DNA was amplified from pHN127 for all 3 plasmids using 5’ 0714_S301A and 3’ CDR20291_0714. These two fragments were inserted using Gibson assembly into pJS116 at the *Not*I and *Xho*I sites [68].

The CD630Δ*erm sspA* gene and promoter region were amplified from CD630Δ*erm* with the primers 5’sspA_MTL and 3’ sspA.pJS116. This fragment was inserted into pMTL84151 at the *Not*I and *Xho*I sites using Gibson assembly, generating pHN152 [68].

For pHN153, the CD630Δ*erm sspA* gene and promoter region were amplified with 5’sspA_MTL and 3’ sspAsspB. The CD630Δ*erm sspB* gene and promoter region were amplified with 5’ sspAsspB and 3’sspBpJS116. These fragments were inserted into pMTL84151 at the *Not*I and *Xho*I sites using Gibson assembly [68].

The CD630Δ*erm sspB* gene and promoter region were amplified using 5’ sspB UP and 3’sspBpJS116. This fragment was inserted into pHN149 at the *Not*I and *Xho*I sites using Gibson assembly to generate pHN176 [68].

The luciferase plasmids pHN209 through pHN211 were generated by amplifying the xylose promoter region from pIA33 using primers 5’ xylR_pHN149 and 3’ PxylR_spoIVB2. The *spoIVB2* gene fragments were amplified with primers 5’ spoIVB2_PxylR and 3’ spoIVB2_luciferase from R20291 for pHN209, HNN19 for pHN210, and HNN33 for pHN211. The *bitLuc* gene fragment with a *ssrA* tag was amplified from pMB81 with primers 5’ luciferase_spoIVB2 and 3’ luciferase_ssrA_pHN149. These 3 fragments were cloned into pHN149 at the *Not*I and *Xho*I restriction sites using Gibson assembly [68]. For the control luciferase plasmids, pHN212–213, the xylose promoter region was amplified from pIA33 using primers 5’ xylR_pHN149 and 3’ PxylR_bitLuc. For pHN212, the *bitLuc* gene portion with a *ssrA* tag was amplified from pMB81 with primers 5’ bitLuc_PxylR and 3’ luciferase_ssrA_pHN149. For pHN213, the *bitLuc* gene was amplified from pMB81 with primers 5’ bitLuc_PxylR and 3’ luciferase_pHN149. The xylose promoter fragment and the luciferase fragments were cloned into pHN149 at the *Not*I and *Xho*I restriction sites using Gibson assembly [68].

pHN220 was generated by amplifying the *sspA* promoter region from *C. difficile* R20291 with primers 5’sspA_MTL and 3’PsspA_BS49. The *sspA* gene was amplified from *B. subtillis* BS49 with primers 5’ sspA_BS49 and 3’ sspA_BS49. These fragments were put into the pHN149 backbone at the *Not*I and *Xho*I sites by Gibson assembly [68].

To generate pHN208, the promoter region through F36 of *CDR20291_0714* was amplified with 5’ CDR20291_0714 and 3’ spoIVB2 F36F, while the F36 through the end of *CDR20291_0714* was amplified with 5’ spoIVB2 F36F and 3’ CDR20291_0714, both using pHN127 as the DNA template. These fragments were inserted by Gibson assembly into the pHN149 plasmid backbone at the *Not*I and *Xho*I sites [68]. pHN218 was generated by amplifying from the pHN127 template DNA *CDR20291_0714* promoter region through F37 with primers 5’ CDR20291_0714 and 3’ spoIVB2 F37.UUA and the fragment with F37 through the end of the gene was amplified by 5’ spoIVB2 F37.UUA and 3’ CDR20291_0714. These fragments were inserted in pHN149 at the *Not*I and *Xho*I sites through Gibson assembly [68]. Similarly, pHN219 was generated but used 5’ CDR20291_0714 with 3’ spoIVB2 F37.UUG for the first fragment and 5’ spoIVB2 F37.UUG and 3’ CDR0291_0714 for the second fragment, both with pHN127 as the template DNA. These were also inserted by Gibson assembly into pHN149 at the *Not*I and *Xho*I sites [68].

All plasmids were sequenced to confirm the correct sequence of the inserts.

### Conjugations:

For conjugations between *C. difficile* and *E. coli* HB101 pRK24, 5 mL of LB supplemented with chloramphenicol and ampicillin was inoculated with a colony from the HB101 pRK24 transformation. Concurrently, *C. difficile* strains were cultured in 5 mL BHIS broth. After approximately 16 hours of incubation, 1 mL of *C. difficile* culture was heat shocked at 52 °C for 5 minutes, in the anaerobic chamber, and then allowed to cool. While heat shocking, 1 mL of *E. coli* culture was centrifuged at 2,348 × g for 5 minutes and the supernatant poured off. The *E. coli* pellets were passed into the chamber and resuspended with the cooled *C. difficile* culture. 20 μL spots were plated onto BHI. The next day, growth was scraped into 1 mL BHIS broth and distributed onto BHIS plates supplemented with thiamphenicol, kanamycin, and cycloserine (TKC) or TKC plus lincomycin (TKLC) for the 2-plasmid CRISPR system.

For conjugations between *C. difficile* and *B. subtilis* BS49, the plasmids generated in DH5α were used to transform *E. coli* MB3436 (a *recA*^+^ strain of *E. coli*) and plasmid purified. This plasmid preparation was then used to transform BS49. *C. difficile* was cultured in 5 mL BHIS broth overnight. After approximately 16 hours, the *C. difficile* culture was back diluted 1:20 and grown for 4 hours. *B. subtilis* was grown for 4 hours in 5 mL BHIS broth supplemented with chloramphenicol and tetracycline. After incubation, the *B. subtilis* cultures were passed into the chamber and 100 μL of BS49 and 100 μL of *C. difficile* were spread onto TY plates. The next day, the growth was resuspended in 1 mL of BHIS broth and distributed between BHIS plates with thiamphenicol and kanamycin. Colonies were screened by streaking colonies onto BHIS supplemented with thiamphenicol and kanamycin and to BHIS supplemented with tetracycline to identify isolates that were tet-sensitive (do not contain the *Tn*916 transposon).

PCR was used to confirm strains and plasmids in each conjugate.

### CRISPR induction:

For induction, colonies were passaged on TY agar supplemented with 1% xylose and thiamphenicol [42, 69]. Mutants were detected by PCR and the plasmid was cured by heat shocking overnight cultures and isolating colonies that had lost their antibiotic resistance.

Induction of R20291 pHN138 resulted in the *C. difficile* CD630Δ*erm* Δ*sspA* mutant, HNN45. R20291 pHN132 induction resulted in the *C. difficile* CD630Δ*erm* Δ*sspB* mutant, HNN43. To generate the *C. difficile* CD630Δ*erm* Δ*sspA* Δ*sspB* strain, the pHN132 vector was induced in HNN45, resulting in HNN46. The *C. difficile CDR20291_0714* (*spoIVB2*) mutant HNN49 was produced from induction of R20291 pHN157.

### Phase contrast imaging:

The strains were inoculated onto 70:30 sporulation media and incubated for 6 days. After, the samples were fixed in a 4% paraformaldehyde and 2% glutaraldehyde solution in 1x PBS. The samples were imaged on a Leica DM6B microscope at the Texas A&M University Microscopy and Imaging Center Core Facility (RRID:SCR_022128).

### TEM:

For sporulating cells, the relevant strains were incubated in the anaerobic chamber on sporulation media for 6 days, and then the growth scraped with an inoculation loop into 1,950 μL of fixative (5% glutaraldehyde, 2% acrolein in 0.05 M HEPES, pH 7.4) in a 2.0 mL microcentrifuge tube. The samples were incubated in the fixative overnight at 4 °C. The following day, the fixed samples were centrifuged for 5 min at >14,000 × g, and post-fixed and stained for 2 hours with 1% osmium tetroxide in 0.05 M HEPES, pH 7.4.

The samples were then centrifuged and washed with water 5 times, and dehydrated with acetone, using the following protocol: 15 minutes in 30%, 50%, 70%, 90% acetone each, then 100% acetone 3 changes, 30 minutes each. At the final wash step, a small amount of acetone, barely covering the pellets, was retained to avoid rehydration of the samples. The samples were then infiltrated with modified Spurr’s resin (Quetol ERL 4221 resin, Electron Microscopy Sciences, RT 14300) in a Pelco Biowave processor (Ted Pella, Inc.), as follows: 1:1 acetone:resin for 10 minutes at 200 W – no vacuum, 1:1 acetone:resin for 5 minutes at 200 W – vacuum 20’’ Hg (vacuum cycles with open sample container caps), 1:2 acetone:resin for 5 minutes at 200 W – vacuum 20’’ Hg, 4 × 100% resin for 5 minutes at 200 W – vacuum 20’’ Hg.

The resin was then removed, and the sample fragments transferred to BEEM conical tip capsules prefilled with a small amount of fresh resin, resin added to fill the capsule, and capsules left to stand upright for 30 minutes to ensure that the samples sank to the bottom. The samples were then polymerized at 65 °C for 48 hours in the oven, then left at RT for 24 hours before sectioning. 70–80 nm samples were sectioned by Leica UC / FC7 ultra-microtome (Leica Microsystems), deposited onto 300 mesh copper grids, stained with uranyl acetate / lead citrate and imaged. All ultrathin TEM sections were imaged on JEOL 1200 EX TEM (JEOL, Ltd.) at 100 kV, images were recorded on SIA-15C CCD (Scientific Instruments and Applications) camera at the resolution of 2721 × 3233 pixels using MaxImDL software (Diffraction Limited). Images were subsequently adjusted for brightness / contrast using Fiji [70]. All equipment used is located at Texas A&M University Microscopy and Imaging Center Core Facility (RRID:SCR_022128).

### Sporulation Assay:

Sporulation assays were completed as previously described [42]. Briefly, 70:30 plates were inoculated with the indicated strains and grown for 48 hours. ¼ of the plate was harvested into 1 mL of PBS. 500 μL was transferred into 500 μL of BHIS and this was serially diluted in BHIS and plated onto BHIS to enumerate vegetative cells. The other 500 μL of culture was treated for 20 minutes with 300 μL of 100% EtOH and 200 μL of dH_2_O to make a 30% final solution. After incubation, the samples were serially diluted into PBS + 0.1% TA and plated onto BHIS supplemented with TA to enumerate spores. The CFUs derived from spores were divided by the total cell count (vegetative cells + spores) and multiplied by 100.

### Spore Purification:

Spores were purified as previously described [46, 71]. Briefly, the cultures from 70:30 agar medium were scraped into 1 mL of dH_2_O and left overnight at 4°C. The next day, the pellets were resuspended and centrifuged for 1 minute at max speed. The upper layer of cell debris was removed and the sample was resuspended in 1 mL dH_2_O. Again, the tubes were centrifuged and the upper layer removed. This was repeated approximately 5 times until the spore pellet was relatively free of debris. The 1 mL of spores in dH_2_O was loaded onto 50% sucrose and centrifuged at 4,000 × g for 20 minutes 4°C. The spore pellet was then washed as described above approximately 5 times and then stored at 4°C until future use.

### UV exposure:

UV experiments were performed as previously described [42]. Briefly, 1×10^8^ spores / mL in PBS were treated for 10 minutes with constant agitation. The T_0_ and T_10_ samples were serially diluted and plated onto rich medium containing germinant taurocholic acid (TA). Treated spore counts were normalized to untreated and then this ratio was normalized to the ratio for wild type spores.

### EMS treatment:

For EMS treatment, the HNN04 or HNN05 strains with pJS116 were used to help prevent contamination by providing antibiotic selection. Overnight cultures were back diluted to OD_600_ 0.05 in 15 mL of BHIS + Tm [44, 45]. The cultures were grown until OD_600_ of 0.5. The culture was split into 2 tubes of 5 mL, each. One tube served as the negative control and one tube was treated with 1% EMS. The cultures were grown for 3 hours with vigorous shaking every 30 minutes (to keep the EMS in solution). The cultures were passed out of the chamber and centrifuged at 3,000 × g for 10 minutes, passed into the chamber, decanted, resuspended with 10 mL BHIS to wash and then passed out and centrifuged again. This wash step was repeated 1 more time for a total of 2 washes. After the second wash, the cell pellet was resuspended with 1 mL of BHIS and deposited into 39 mL BHIS + Tm to recover overnight.

The next day, to determine mutagenesis rates, 10 μL, 25 μL, and 50 μL volumes were each plated onto BHIS rifampicin agar and CFUs were counted after 24 – 48 hours. From the EMS (+) culture, 50 μL was plated onto 20 BHIS Tm5 agar plates and left in the chamber to incubate for 5 days. For the EMS (−) culture, a whole genome prep was performed as described below.

After the incubation period, the plates were scraped into individual tubes with 1 mL dH_2_O and left overnight at 4 °C. The tubes were purified to remove cell debris as done with spore purification described above. The samples were combined to one tube and heated at 65 °C for 1 hour, with intermittent vortexing. The sample was then distributed between 20 BHIS Tm5 plates for another round of incubation. This enrichment step was completed 3 times before isolates were selected and PCR was used to confirm the genotype (to confirm that wildtype contamination did not occur during the selection). After confirmation, the samples were plated onto 70:30 Tm5 and incubated for 5 days. These samples were then checked under a phase contrast microscope for spores. Genomic DNA was purified from samples that had spores and sent for whole genome resequencing at Microbial Genome Sequencing Center (MiGS; Pittsburgh, PA).

### Whole genome preparation:

4 tubes of 10 mL each were inoculated overnight for approximately 18 hours (or the 40 mL of culture from EMS (−) strains were used). The next day, the samples were centrifuged at 4,000 × g for 10 minutes, 4 °C. They were decanted, then resuspended with 1 mL TE buffer (10 mM Tris-HCl, 1 mM EDTA). Samples were centrifuged again, decanted, and resuspended with 200 μL of genomic DNA solution (34% sucrose in TE buffer) and transferred to a 2 mL Eppendorf tube (for each strain, the 4 tubes are kept separate). The tubes were incubated at 37°C for 2 hours. Then, 100 μL of 20% Sarkosyl and 15 μL of 10 mg / mL RNase A were added to the sample and incubated at 37 °C for 30 minutes. After this incubation, 15 μL of proteinase K solution was added and incubated 37 °C for 30 minutes at. The samples were brought up to 600 μL with TE buffer.

600 μL of 25:24:1 phenol/chloroform/isoamyl alcohol was added to the samples and were rocked gently for 20 minutes. After the incubation, the samples were centrifuged for 10 minutes at max speed. The upper layer was transferred to a new tube with a cut pipette tip (so as not to shear the DNA) and 600 μL chloroform was added to the sample and rocked for another 20 minutes. The centrifugation, sample transfer, and chloroform treatment were repeated for a total of 3 times. After which, the upper phase was transferred to a new tube and precipitated at −20 °C overnight with 50 μL of 3 M sodium acetate, pH 5.2, and 3 volumes of cold 95% ethanol.

After precipitation, one tube from each strain was centrifuged 15 minutes at max speed, 4 °C. The supernatant was discarded and the solution from the second tube was transferred to the tube with the DNA pellet and centrifuged again. This was repeated until the DNA from all 4 tubes was combined into one pellet. The DNA pellet was washed with 500 μL of 70% ethanol and centrifuged again. The samples were decanted and allowed to dry at room temperature until all of the ethanol was evaporated (approximately 60 – 90 minutes). After drying, 500 μL of either dH_2_O or TE buffer was added, and the samples were rocked overnight to allow the pellets to dissolve.

### RNA extraction and processing:

Strains were plated onto 70:30 media for 11 hours before extraction. RNA extraction was performed using the FastRNA Pro Blue Kit (MP Biomedicals, Solon, OH). Briefly, the culture was scraped into 1 mL of PBS and centrifuged 2,348 × g for 5 minutes. The pellet was resuspended in 1 mL of RNApro solution and transferred to the provided tubes with lysing Matrix B. The cells were lysed in an MP FastPrep-24 bead beater for 40 seconds on and 20 seconds off for a total of 2 rounds. Further processing followed the FastRNA Pro Blue Kit protocol except that the RNA was precipitated overnight, and the remainder of the protocol was continued the next day.

Contaminating DNA was removed using the TURBO DNA-free kit (Invitrogen, Waltham, MA). 10 μg of RNA was treated 3 times with DNase following the protocol provided in the kit. The RNA was precipitated at −20 °C overnight with 0.1 volume of 3 M sodium acetate, 5 μg of glycogen, and an equal volume of 100% ethanol. RNA was recovered by centrifuging at 13,000 × g at 4 °C for 30 minutes. The pellet was washed 2 times with 70% cold ethanol. The pellet was air-dried at room temperature and then resuspended in dH_2_O.

cDNA was generated using the Superscript III First-Strand Synthesis System (Invitrogen, Waltham, MA) reagents and protocol.

### q-RT-PCR:

qPCR was performed with PowerUP SYBR Green Master Mix (Applied Biosystems, Waltham, MA) according to provided protocol on an Applied Biosystems QuantStudio 6 Flex Real-Time PCR system. Primers used are as follows: *rpoA*: 5’ rpoA & 3’ rpoA; *sspA*: 5’ sspA_qPCR & 3’ sspA_qPCR; *sspB*: 5’ sspB_qPCR & 3’ sspB_qPCR; *sleC*: 5’sleC_qPCR & 3’sleC_qPCR; *spoVT*: 5’ spoVT_qPCR & 3’ spoVT_qPCR; *pdaA*: 5’ pdaA_qPCR & 3’ pdaA_qPCR; *spoIVA*: 5’spoIVA_qPCR & 3’spoIVA_qPCR; *spoIVB*: 5’ spoIVB_qPCR_1 & 3’ spoIVB_qPCR_1; *spoIVB2*: 5’ spoIVB2_qPCR_1 & 3’ spoIVB2_qPCR_1; *spoIIP*: 5’ spoIIP_qPCR_1 & 3’ spoIIP_qPCR_1; *dpaA*: 5’ dpaA_qPCR & 3’ dpaA_qPCR; *spoVAC*: 5’ spoVAC_qPCR & 3’ spoVAC_qPCR; *spoVAD*: 5’ spoVAD_qPCR & 3’ spoVAD_qPCR; *spoVAE*: 5’ spoVAE_qPCR & 3’ spoVAE_qPCR.

Analysis was performed by the ΔΔCT method with comparison to internal control *rpoA* and then mutant strains compared to WT (R20291) [72].

### Luciferase Assays:

Overnight cultures were back diluted to OD_600_ = 0.05 in BHIS supplemented with thiamphenicol. The cultures were grown to mid-log and split into two, where one was induced with 0.5% D-xylose and one was left uninduced. After 30 minutes, the OD_600_ was recorded and the cultures were used for the Nano-Glo Luciferase assay (Promega, Madison, WI). Briefly, 100 μL of culture was put into a standard Optiplate White bottom 96 well plate. 20 μL of buffer/substrate mixture, prepared as per the kit instructions, was added to the culture. The plate was shaken for 3 minutes before the RFU was determined. The RFU was normalized to the OD_600_ for the uninduced samples and induced samples. Then the ratio of uninduced was subtracted from the ratio for the induced samples.[51, 52]

For each trial, 2 technical replicates were measured in different positions in the 96 well plate, due to some variation in measurements based on location within the plates.

## Figures and Tables

**Table 1:** Shorten list of potential suppressor mutations **Mutations identified in suppressor strains.** The mutations that could potentially suppress the sporulation defect in EMS treated isolates.

Gene	Function	Isolates	Mutation
*rpoB*	RNA polymerase beta subunit	HNN37	P893S
*rpoC*	RNA polymerase beta’ subunit	HNN40	H94Y
*rpoC*	RNA polymerase beta’ subunit	HNN32	W225 stp
*rpoC*	RNA polymerase beta’ subunit	HNN40, HNN22	S315F
*rpoA*	RNA polymerase alpha subunit	HNN51, HNN19	S281F
*CDR20291_0714*	Stage IV sporulation protein	HNN19	A20T
*CDR20291_0714*	Stage IV sporulation protein	HNN33, HNN35, HNN37, HNN38, HNN41, HNN48	F37F; Synonymous
*sigG*	Forespore sporulation sigma factor	HNN39	M97I
*sspA*	Small acid soluble protein	HNN26, HNN28	V52G; Reversion
*spoVT*	Stage V sporulation protein	HNN51	P39S

## Importance

*Clostridioides difficile* is easily spread through the production of highly resistant spores. Understanding how spores are formed could yield valuable insight into how the sporulation process can be halted to render spores that are sensitive to cleaning methods. Here, we identify another protein involved in the sporulation process that is seemingly controlled by the small acid-soluble proteins (SASPs). This discovery allows us to better understand how the *C. difficile* SASPs may bind to specific sites on the genome to regulate gene expression.

## References

[1] McDonald LC, Gerding DN, Johnson S, Bakken JS, Carroll KC, Coffin SE, Clinical Practice Guidelines for Clostridium difficile Infection in Adults and Children: 2017 Update by the Infectious Diseases Society of America (IDSA) and Society for Healthcare Epidemiology of America (SHEA). Clin Infect Dis. 2018;66(7):987–94. doi: 10.1093/cid/ciy149. 29562266

[2] Voth DE, Ballard JD. Clostridium difficile toxins: mechanism of action and role in disease. Clin Microbiol Rev. 2005;18(2):247–63. doi: 10.1128/CMR.18.2.247-263.2005.. 15831824 PMC1082799

[3] Smits WK, Lyras D, Lacy DB, Wilcox MH, Kuijper EJ. Clostridium difficile infection. Nat Rev Dis Primers. 2016;2:16020. doi: 10.1038/nrdp.2016.20. 27158839 PMC5453186

[4] Jump RL, Pultz MJ, Donskey CJ. Vegetative *Clostridium difficile* survives in room air on moist surfaces and in gastric contents with reduced acidity: a potential mechanism to explain the association between proton pump inhibitors and *C. difficile*-associated diarrhea? Antimicrob Agents Chemother. 2007;51(8):2883–7. doi: 10.1128/AAC.01443-06. 17562803 PMC1932506

[5] Deakin LJ, Clare S, Fagan RP, Dawson LF, Pickard DJ, West MR, The *Clostridium difficile spo0A* gene is a persistence and transmission factor. Infect Immun. 2012;80(8):2704–11. Epub 2012/05/23. doi: 10.1128/IAI.00147-12. 22615253 PMC3434595

[6] Seekatz AM, Young VB. *Clostridium difficile* and the microbiota. J Clin Invest. 2014;124(10):4182–9. Epub 20140718. doi: 10.1172/JCI72336. 25036699 PMC4191019

[7] Lawley TD, Clare S, Walker AW, Goulding D, Stabler RA, Croucher N, Antibiotic treatment of *Clostridium difficile* carrier mice triggers a supershedder state, spore-mediated transmission, and severe disease in immunocompromised hosts. Infect Immun. 2009;77(9):3661–9. doi: 10.1128/IAI.00558-09. 19564382 PMC2737984

[8] Paredes-Sabja D, Shen A, Sorg JA. *Clostridium difficile* spore biology: sporulation, germination, and spore structural proteins. Trends Microbiol. 2014;22(7):406–16. doi: 10.1016/j.tim.2014.04.003. 24814671 PMC4098856

[9] Permpoonpattana P, Tolls EH, Nadem R, Tan S, Brisson A, Cutting SM. Surface layers of *Clostridium difficile* endospores. J Bacteriol. 2011;193(23):6461–70. Epub 2011/09/29. doi: 10.1128/JB.05182–11 JB.05182–11 [pii]. 21949071 10.1128/JB.05182-11 PMC3232898

[10] Baloh MaS J.A.. *Clostridioides difficile* SpoVAD and SpoVAE Interact and Are Required for Dipicolinic Acid Uptake into Spores. J Bacteriol. 2021;203(21). doi: 10.1128/JB.00394-21. PMC8508128 34424035

[11] Setlow B, Magill N, Febbroriello P, Nakhimovsky L, Koppel DE, Setlow P. Condensation of the forespore nucleoid early in sporulation of Bacillus species. J Bacteriol. 1991;173(19):6270–8. doi: 10.1128/jb.173.19.6270-6278.1991. 1917859 PMC208380

[12] Setlow P. Spore resistance properties. Microbiol Spectr. 2014;2(5). doi: 10.1128/microbiolspec.TBS-0003-2012. 26104355

[13] Setlow P. I will survive: DNA protection in bacterial spores. Trends Microbiol. 2007;15(4):172–80. doi: 10.1016/j.tim.2007.02.004. 17336071

[14] Burns DA, Heap JT, Minton NP. SleC is essential for germination of *Clostridium difficile* spores in nutrient-rich medium supplemented with the bile salt taurocholate. J Bacteriol. 2010;192(3):657–64. doi: 10.1128/jb.01209-09. 19933358 PMC2812441

[15] Gutelius D, Hokeness K, Logan SM, Reid CW. Functional analysis of SleC from *Clostridium difficile*: an essential lytic transglycosylase involved in spore germination. Microbiology. 2014;160(Pt 1):209–16. doi: 10.1099/mic.0.072454-0. 24140647 PMC3917228

[16] Coullon H, Candela T. *Clostridioides difficile* peptidoglycan modifications. Curr Opin Microbiol. 2022;65:156–61. Epub 20211206. doi: 10.1016/j.mib.2021.11.010. 34883390

[17] Driks AaE P.. The spore coat microbiol Spectr. 2016;4(2). doi: 10.1128/microbiolspec.TBS. 27227299

[18] Driks A. Surface appendages of bacterial spores. Molecular Microbiology. 2007;63(3):623–5. 17302795 10.1111/j.1365-2958.2006.05564.x

[19] Permpoonpattana P, Phetcharaburanin J, Mikelsone A, Dembek M, Tan S, Brisson MC, Functional characterization of *Clostridium difficile* spore coat proteins. J Bacteriol. 2013;195(7):1492–503. Epub 2013/01/22. doi: 10.1128/JB.02104-12. 23335421 PMC3624542

[20] Pizarro-Guajardo M, Calderon-Romero P, Castro-Cordova P, Mora-Uribe P, Paredes-Sabja D. Ultrastructural variability of the exosporium layer of *Clostridium difficile* spores Appl Environ Microbiol. 2016;82(7):2202–9. doi: 10.1111/mmi.12611. 26850296 PMC4807528

[21] Zeng J, Wang H, Dong M, Tian GB. *Clostridioides difficile* spore: coat assembly and formation. Emerg Microbes Infect. 2022;11(1):2340–9. doi: 10.1080/22221751.2022.2119168. 36032037 PMC9542656

[22] de Hoon MJ, Eichenberger P, Vitkup D. Hierarchical evolution of the bacterial sporulation network. Current biology : CB. 2010;20(17):R735–45. Epub 2010/09/14. doi: 10.1016/j.cub.2010.06.031. 20833318 PMC2944226

[23] Talukdar PK, Olguin-Araneda V, Alnoman M, Paredes-Sabja D, Sarker MR. Updates on the sporulation process in Clostridium species. Res Microbiol. 2015;166(4):225–35. doi: 10.1016/j.resmic.2014.12.001. 25541348

[24] Paredes-Sabja D, Torres JA, Setlow P, Sarker MR. *Clostridium perfringens* spore germination: characterization of germinants and their receptors. J Bacteriol. 2008;190(4):1190–201. doi: 10.1128/jb.01748-07. 18083820 PMC2238220

[25] Lee CD, Rizvi A, Edwards AN, DiCandia MA, Vargas Cuebas GG, Monteiro MP, Genetic mechanisms governing sporulation initiation in Clostridioides difficile. Curr Opin Microbiol. 2022;66:32–8. Epub 20211218. doi: 10.1016/j.mib.2021.12.001. 34933206 PMC9064876

[26] DiCandia MA, Edwards AN, Jones JB, Swaim GL, Mills BD, McBride SM. Identification of Functional Spo0A Residues Critical for Sporulation in Clostridioides difficile. J Mol Biol. 2022;434(13):167641. Epub 20220518. doi: 10.1016/j.jmb.2022.167641.. 35597553 PMC9327077

[27] Fimlaid KA, Bond JP, Schutz KC, Putnam EE, Leung JM, Lawley TD, Global analysis of the sporulation pathway of Clostridium difficile. PLoS Genet. 2013;9(8):e1003660. Epub 2013/08/21. doi: 10.1371/journal.pgen.1003660PGENETICS-D-12-03167 [pii]. 23950727 PMC3738446

[28] Fimlaid KA, Shen A. Diverse mechanisms regulate sporulation sigma factor activity in the Firmicutes. Curr Opin Microbiol. 2015;24:88–95. doi: 10.1016/j.mib.2015.01.006. 25646759 PMC4380625

[29] Small Setlow P., acid-soluble spore proteins of *Bacillus* species: structure, synthesis, genetics, function, and degradation. Ann Rev Microbial 1988;42. 10.1146/annurev.mi.42.100188.001535 3059997

[30] Mason J, Setlow P. Essential role of small, acid-soluble spore proteins in resistance of *Bacillus subtilis* spores to UV light. Journal of Bacteriology 1986;167:174–8. 3087950 10.1128/jb.167.1.174-178.1986 PMC212857

[31] Raju D, Setlow P, Sarker MR. Antisense-RNA-mediated decreased synthesis of small, acid-soluble spore proteins leads to decreased resistance of *Clostridium perfringens* spores to moist heat and UV radiation. Appl Environ Microbiol. 2007;73(7):2048–53. Epub 2007/01/30. doi: 10.1128/AEM.02500-06. 17259355 PMC1855649

[32] Li J, McClane BA. A novel small acid soluble protein variant is important for spore resistance of most Clostridium perfringens food poisoning isolates. PLoS Pathog. 2008;4(5):e1000056. Epub 20080502. doi: 10.1371/journal.ppat.1000056. 18451983 PMC2323104

[33] Li J, Paredes-Sabja D, Sarker MR, McClane BA. Further characterization of Clostridium perfringens small acid soluble protein-4 (Ssp4) properties and expression. PLoS One. 2009;4(7):e6249. Epub 20090717. doi: 10.1371/journal.pone.0006249. 19609432 PMC2706996

[34] Mohr SC, Sokolov NVHA, He C, Setlow P. Binding of small acid-soluble spore proteins from *Bacillus subtilis* changes the conformation of DNA from B to A. Proc Natl Acad Sci. 1991;88:77–81. 1898779 10.1073/pnas.88.1.77 PMC50751

[35] Seog Lee K, Bumbaca D, Kosman J, Setlow P, Jedrzejas MJ. Structure of a protein–DNA complex essential for DNA protection in spores of *Bacillus* species. PNAS. 2007;105(8). 10.1073/pnas.0708244105 PMC2268541 18287075

[36] Griffith J, Makhov A, Santiago-Larat L, Setlow P. Electron microscopic studies of the interaction between a *Bacillus subtilis* α/β-type small, acid-soluble spore protein with DNA: protein binding is cooperative, stiffens the DNA, and induces negative supercoiling. Proc Natl Acad Sci. 1994;91:8224–8. 8058784 10.1073/pnas.91.17.8224 PMC44578

[37] Setlow B, Sun D, Setlow P. Interaction between DNA and α/β-type small, acid-soluble spore proteins: a new class of DNA-binding protein. J Bacteriol. 1992. 10.1128/jb.174.7.2312-2322.1992 PMC205853 1313001

[38] Setlow P. Resistance of spores of *Bacillus* species to ultraviolet light. Environmental and Molecular Mutagenesis 2001;38:97–104. 11746741 10.1002/em.1058

[39] Munakata N, Ruper CS. Genetically Controlled Removal of “Spore Photoproduct” from Deoxyribonucleic Acid of Ultraviolet-Irradiated Bacillus subtilis Spores. J Bacteriol. 1972;111(1):192–8. 4204907 10.1128/jb.111.1.192-198.1972 PMC251257

[40] Yang L, Li L. Spore photoproduct lyase: the known, the controversial, and the unknown. J Biol Chem. 2015;290(7):4003–9. Epub 2014/12/06. doi: 10.1074/jbc.R114.573675. 25477522 PMC4326811

[41] Sanchez-Salas J-L, Santiago-Lara ML, Sussman MD, Setlow P. Properties of *Bacillus megaterium* and *Bacillus subtilis* mutants which lack the protease that degrades small, acid-soluble proteins during spore germination. J Bacteriol. 1992;174(3):804–14. 10.1128/jb.174.3.807-814.1992 PMC206157 1732215

[42] Nerber HN, Sorg JA. The small acid-soluble proteins of *Clostridioides difficile* are important for UV resistance and serve as a check point for sporulation. PLoS Pathog. 2021;17(9):e1009516. Epub 20210908. doi: 10.1371/journal.ppat.1009516. 34496003 PMC8452069

[43] Leyva-Illades JF, Setlow B, Sarker MR, Setlow P. Effect of a small, acid-soluble spore protein from *Clostridium perfringens* on the resistance properties of *Bacillus subtilis* spores. J Bacteriol. 2007;189(21):7927–31. Epub 2007/09/04. doi: 10.1128/JB.01179-07. 17766414 PMC2168745

[44] Shrestha R, Cochran AM, Sorg JA. The requirement for co-germinants during Clostridium difficile spore germination is influenced by mutations in yabG and cspA. PLoS Pathog. 2019;15(4):e1007681. doi: 10.1371/journal.ppat.1007681. 30943268 PMC6464247

[45] Francis MB, Allen CA, Shrestha R, Sorg JA. Bile acid recognition by the Clostridium difficile germinant receptor, CspC, is important for establishing infection. PLoS Pathog. 2013;9(5):e1003356. doi: 10.1371/journal.ppat.1003356.. 23675301 PMC3649964

[46] Sorg JA, Sonenshein AL. Bile salts and glycine as cogerminants for *Clostridium difficile* spores. J Bacteriol. 2008;190(7):2505–12. doi: 10.1128/JB.01765-07. 18245298 PMC2293200

[47] Saujet L, Pereira FC, Serrano M, Soutourina O, Monot M, Shelyakin PV, Genome-wide analysis of cell type-specific gene transcription during spore formation in *Clostridium difficile*. PLoS Genet. 2013;9(10):e1003756. doi: 10.1371/journal.pgen.1003756. 24098137 PMC3789822

[48] Eijlander RT, Holsappel S, de Jong A, Ghosh A, Christie G, Kuipers OP. SpoVT: From Fine-Tuning Regulator in Bacillus subtilis to Essential Sporulation Protein in Bacillus cereus. Front Microbiol. 2016;7:1607. Epub 2016/10/30. doi: 10.3389/fmicb.2016.01607. 27790204 PMC5061766

[49] Hoa NT, Brannigan JA, Cutting SM. The *Bacillus subtilis* signaling protein SpoIVB defines a new family of serine peptidases. J Bacteriol. 2002;184(1):191–9. Epub 2001/12/14. doi: 10.1128/JB.184.1.191–199.2002. 11741860 10.1128/JB.184.1.191-199.2002 PMC134772

[50] Baloh M, Sorg JA. *Clostridioides difficile* SpoVAD and SpoVAE interact and are required for dipicolinic acid uptake into spores. J Bacteriol. 2021;203(21):e0039421. Epub 2021/08/24. doi: 10.1128/JB.00394–21. 34424035 10.1128/JB.00394-21 PMC8508128

[51] Oliveira Paiva AM, Friggen AH, Hossein-Javaheri S, Smits WK. The Signal Sequence of the Abundant Extracellular Metalloprotease PPEP-1 Can Be Used to Secrete Synthetic Reporter Proteins in *Clostridium difficile*. ACS Synth Biol. 2016;5(12):1376–82. doi: 10.1021/acssynbio.6b00104. 27333161

[52] Oliveira Paiva AM, Friggen AH, Qin L, Douwes R, Dame RT, Smits WK. The Bacterial Chromatin Protein HupA Can Remodel DNA and Associates with the Nucleoid in *Clostridium difficile*. J Mol Biol. 2019;431(4):653–72. Epub 2019/01/12. doi: 10.1016/j.jmb.2019.01.001.. 30633871

[53] Lavey NP, Shadid T, Ballard JD, Duerfeldt AS. Clostridium difficile ClpP Homologues are Capable of Uncoupled Activity and Exhibit Different Levels of Susceptibility to Acyldepsipeptide Modulation. ACS Infect Dis. 2019;5(1):79–89. Epub 20181126. doi: 10.1021/acsinfecdis.8b00199.. 30411608 PMC6497155

[54] Tovar-Rojo F, Setlow P. Effects of Mutant Small, Acid-Soluble Spore Proteins from *Bacillus subtilis* on DNA In Vivo and In Vitro. J Bacteriol. 1991;173(15):4827–35. 1906873 10.1128/jb.173.15.4827-4835.1991 PMC208162

[55] Diaz OR, Sayer CV, Popham DL, Shen A. Clostridium difficile lipoprotein GerS is required for cortex modification and thus spore germination. mSphere. 2018;3(3). doi: 10.1128/mSphere.00205-18. PMC6021603 29950380

[56] Coullon H, Rifflet A, Wheeler R, Janoir C, Boneca IG, Candela T. N-Deacetylases required for muramic-delta-lactam production are involved in *Clostridium difficile* sporulation, germination, and heat resistance. J Biol Chem. 2018;293(47):18040–54. Epub 2018/09/30. doi: 10.1074/jbc.RA118.004273. 30266804 PMC6254358

[57] Feliciano CA, Eckenroth BE, Diaz OR, Doublié S, Shen A. A lipoprotein allosterically activates the CwlD amidase during Clostridioides difficile spore formation. PLoS Genet. 2021;17(9). doi: 10.1101/2021.06.21.449279. 10.1371/journal.pgen.1009791 PMC8496864 34570752

[58] Francis MB, Sorg JA. Dipicolinic acid release by germinating Clostridium difficile spores occurs through a mechanosensing mechanism. mSphere. 2016;1(6). doi: 10.1128/mSphere.00306-16.. PMC5156672 27981237

[59] Hoch JA. Regulation of the Phosphorelay and the Initiation of Sporulation in Bacillus subtilis Ann Rev Microbial. 1993;47:441–65. 10.1146/annurev.mi.47.100193.002301 8257105

[60] Dong TC, Cutting SM. SpoIVB-mediated cleavage of SpoIVFA could provide the intercellular signal to activate processing of Pro-s^K^ in *Bacillus subtilis*. Mol Microbiol. 2003;49 (5):1425–34. 12940997 10.1046/j.1365-2958.2003.03651.x

[61] Crawshaw AD, Serrano M, Stanley WA, Henriques AO, Salgado PS. A mother cell-to-forespore channel: current understanding and future challenges. FEMS Microbiol Lett. 2014;358(2):129–36. doi: 10.1111/1574-6968.12554. 25105965

[62] Chiba S, Coleman K, Pogliano K. Impact of membrane fusion and proteolysis on SpoIIQ dynamics and interaction with SpoIIIAH. J Biol Chem. 2007;282(4):2576–86. Epub 20061122. doi: 10.1074/jbc.M606056200. 17121846 PMC2885159

[63] Oke V, Shchepetov M, Cutting SM. SpoIVB has two distinct functions during spore formation in *Bacillus subtilis*. Mol Microbiol. 1997;23(2):223–30. 9044256 10.1046/j.1365-2958.1997.2091573.x

[64] Nakamura Y, Gojobori T, Ikemura T. Codon usage tabulated from the international DNA sequence databases: status for the year 2000. Nucleic Acids Res. 2000;28(292). 10.1093/nar/28.1.292 PMC102460 10592250

[65] Ribis JW, Fimlaid KA, Shen A. Differential requirements for conserved peptidoglycan remodeling enzymes during *Clostridioides difficile* spore formation. Mol Microbiol. 2018;110(3):370–89. doi: 10.1111/mmi.14090.. 30066347 PMC6311989

[66] Setlow B, McGinnis KA, Ragkousi K, Setlow P. Effects of Major Spore-Specific DNA Binding Proteins on *Bacillus subtilis* Sporulation and Spore Properties J Bacteriol. 2000;182(24):6906–12. 11092849 10.1128/jb.182.24.6906-6912.2000 PMC94814

[67] Sorg JA, Dineen SS. Laboratory maintenance of Clostridium difficile. Curr Protoc Microbiol. 2009;Chapter 9:Unit9A 1. doi: 10.1002/9780471729259.mc09a01s12. 19235151

[68] Gibson DG, Young L, Chuang RY, Venter JC, Hutchison CA 3rd, Smith HO. Enzymatic assembly of DNA molecules up to several hundred kilobases. Nat Methods. 2009;6(5):343–5. doi: 10.1038/nmeth.1318.. 19363495

[69] Brehm JN, Sorg JA. Plasmid Sequence and Availability for an Improved Clostridioides difficile CRISPR-Cas9 Mutagenesis System. Microbiol Resour Announc. 2022;11(12):e0083322. Epub 20221107. doi: 10.1128/mra.00833-22.. 36342279 PMC9753633

[70] Schindelin J, Arganda-Carreras I, Frise E, Kaynig V, Longair M, Pietzsch T, Fiji: an open-source platform for biological-image analysis. Nat Methods. 2012;9(7):676–82. Epub 20120628. doi: 10.1038/nmeth.2019. 22743772 10.1038/nmeth.2019 PMC3855844

[71] Francis MB, Allen CA, Sorg JA. Spore cortex hydrolysis precedes dipicolinic acid release during *Clostridium difficile* spore germination. J Bacteriol. 2015;197(14):2276–83. doi: 10.1128/JB.02575–14. 25917906 10.1128/JB.02575-14 PMC4524186

[72] Schmittgen TD, Livak KJ. Analyzing real-time PCR data by the comparative C(T) method. Nature protocols. 2008;3(6):1101–8. doi: 10.1038/nprot.2008.73. 18546601 10.1038/nprot.2008.73

