# Supplementary material for: The small acid-soluble proteins of Clostridioides difficile regulate sporulation in a SpoIVB2-dependent manner
Source: bioRxiv. 2024 Jun 25:2023.05.17.541253. Originally published 2023 May 18. Preprint. [Version 2] doi: 10.1101/2023.05.17.541253 (PMC10245694; doi:10.1101/2023.05.17.541253)
Supplement: Supplement 1 [file media-1.pdf]

Supplement Figure 1. EMS mutagenesis strategy. EMS was added to logarithmically growing cultures and incubated for 3 hours. The resulting cells were spread on sporulation medium (70:30) and incubated for 5 days. Subsequently, the growth was harvested, purified, and then then heat shocked at 65 °C for 1 hour. After heat treatment, the samples were plated onto 70:30 and again incubated for 5 days. This enrichment process was repeated 3 times before individual colonies were isolated, phenotypes confirmed, and DNA sent for whole genome re-sequencing. The figure was generated using BioRender.com.

Supplement Figure 2. Spore yield is reduced with expression of *spoIVB2* in EMS strains*.* Spore yield of the indicated strain was determined as described in Figure 1. pEV indicates an empty vector. A) Strains isolated during EMS. B) Clean strains containing generated *spoIVB2* alleles. All data represents the average of three independent experiments. Statistical analysis by one way ANOVA with Šídák's multiple comparison test. * P<0.05, ** P<0.01, *** P<0.001, **** P<0.0001. B) **a** P<0.0001 in comparison to *C. difficile* Δ*sspA* Δ*sspB*.

Supplement Figure 3. qPCR of mutant strains: Part 1*.* Strains were grown on sporulation medium for 11 hours before RNA extraction. qPCR was performed using SYBR green. Transcripts for the following genes were determined: A) *sspA*, B) *sspB*, C) *spoIVA*, D) *sleC*, E) *pdaA*, F) *spoVT.* Fold change from R20291 was determined with the ΔΔCT method using *rpoA* transcripts as the internal control. All data represents the average of five independent experiments. Statistical analysis by one way ANOVA with Dunnett's multiple comparison test with the mutant strains compared to wild type. * P<0.05, ** P<0.01, *** P<0.001, **** P<0.0001.

Supplement Figure 4. qPCR of mutant strains: Part 2*.* Strains were grown on sporulation medium for 11 hours before RNA extraction. qPCR was performed using SYBR green. Transcripts for the following genes were determined: A) *spoIVB*, B) *spoIVB2*, C) *spoIIP.* Fold change from R20291 was determined with the ΔΔCT method using *rpoA* transcripts as the internal control. All data represents the average of five independent experiments. Statistical analysis by one way ANOVA with Dunnett's multiple comparison test with the mutant strains compared to wild type. * P<0.05, ** P<0.01, *** P<0.001, **** P<0.0001.

Supplement Figure 5. qPCR of mutant strains: Part 3*.* Strains were grown on sporulation medium for 11 hours before RNA extraction. qPCR was performed using SYBR green. Transcripts for the following genes were determined: A) *dpaA*, B) *spoVAC*, C) *spoVAD*, D) *spoVAE.* Fold change from R20291 was determined with the ΔΔCT method using *rpoA* transcripts as the internal control. All data represents the average of five independent experiments. Statistical analysis by one way ANOVA with Dunnett's multiple comparison test with the mutant strains compared to wild type. * P<0.05, ** P<0.01, *** P<0.001, **** P<0.0001.

Supplement Table 3. Mutations found within suppressor strains. The mutations identified in EMS treated suppressor strains are sorted by position within the genome and color-coded based on the isolate containing the mutation.

Supplement Table 1. Primers used in this study.

Primer Name	Sequence
5'sspA_MTL	ttatcaggaaacagctatgaccgcggccgcttagatgaggaaaaactggataa

3'sspA_up	ttatttataactatctgttgctttttccaggttgattaccttccttctgttta
5'sspA_down	aataaattaaacagaaggaaggtaatcaacctggaaaaagcaacagatagt
3' sspA_xylR	tgcaggcttcttatttttatgctagctcgagctattgaacttggaaatgagag
CRISPR_sspA_165	gtgtgctataattaaactgtaaaacgcgtgactaaaaaattagttgaaagttttagagctagaaatagcaagttaaaataaggctagtccgttatcaacttgaaaaagtggcaccgagtcggtgctttttttctatggagaaatctagatcagcatgatgtctgactagacgcgtaagctctgcaactatttttagat
5'traJ	gcgaggaagcggaagagcgcccaatacgcagggccccctgcttcggggtca
3'traJ	aatttatctacaattttttatcctgcagggggcccgatcggtcttgccttg
CRISPR_sspA_135	taattaaactgtaaaggtaccagagaaaatggttatgtagggttttagagctagaaatagcaagttaaaataaggctagtccgttatcaacttgaaaaagtggcaccgagtcggtgctttttttctatggagaaatctagatcagcatgatgtctgactagacgcgtaagctctgcaactat
5' sspB UP	attttttatcaggaaacagctatgaccgcggccgcttttaaaatatcatccatattat
3' sspB UP	tgtcaaaatttactatttattttccagccacctcaaataattagtttatgatg
5' sspB DN	tgttagacatcataaactaattatttgaggtggctggaaaataaatagta
3' sspB_xylR	atgcaggcttcttatttttatgctagctcgagatacttgtctatttttcagtaa
CRISPR_sspB_144	aattaaactgtaaaggtaccagagaaaatggatatgttgggttttagagctagaaatagcaagttaaaataaggctagtccgttatcaacttgaaaaagtggcaccgagtcggtgctttttttctatggagaaatctagatcagcatgatgtctgactagacgcgtaagctctgcaacta
5' CDR20291_0714 UP	ttatcaggaaacagctatgaccgcggccgcccttgatgccctgggtctatac
3' CDR20291_0714 UP	cttatttatttatattatttacaacatccgttgcataaaacacctctttct
5' CDR20291_0714 DN	ttaataagaaagaggtgttttatgcaacggatgttgtaaataatataaataa
3' CDR20291_0714 DN	gcaggcttcttatttttatgctagctcgagggaacagcattaggaagtcc
CDR20291_0714 gRNA 3	gcattcaaggagggggtaccgtatctattttattaaataggttttagagctagaaatagc
3' gRNA_change	ccatctaaaaatagttgcagagcttacgcgtctagtcagacatcatgctgatctag
5' tn916.traJ	tctgcagattacctaataatttatctacattccctttagcctgcttcggggtcattat
5'Tn916ori_gibson	cggaagagcgcccaatacgcagggccctaacatcttctatttttcccaaatc
3' tn916.traJ	cgaaaaaatcgctataatgaccccgaagcaggctaaagggaatgtagataaattattag
5' CDR20291_0714	ttttttatcaggaaacagctatgaccgcggccgctgctatctccttttccttg
3' CDR20291_0714	gtgccaagcttgcatgtctgcaggcctcgagttacaacatccatttaataaatac
3' 0714_S301A	ttattatcttgaactattggagtacctgccataccttgtacaataccacc

5' 0714_S301A	ctcaaactggtggtattgtacaaggtatggcaggtactccaatagttcaag
3' sspA.pJS116	tgccaagcttgcatgtctgcaggcctcgagctatctgttgctttttccag
3' sspAsspB	ggaactgataatatggatgatattttaaaactatctgttgctttttccagccatttg
5' sspAsspB	caaatggctggaaaaagcaacagatagttttaaaatatcatccatattat
3'sspBpJS116	cagtgccaagcttgcatgtctgcaggcctcgagttattttccagccatttgtc
5' rpoA	taaaggtagaggttatgtttctgct
3' rpoA	tttgaccaactcttgtgttttcc
5' sspA_qPCR	caaaagaggctttaaaccaaatgaa
3' sspA_qPCR	attttctcttgcagtaaggtttcctt
5' sspB_qPCR	aacagaacagtagttccagaagcaaa
3' sspB_qPCR	caacatatccattttctctagctgttaag
5'sleC_qPCR	ttgaagcaagacaaggagttccc
3'sleC_qPCR	cgaaaccagtaggaggaggtaatgg
5' spoVT_qPCR	agagaaggagaccctttagagat
3' spoVT_qPCR	ctgttatcaacactccatatcctagt
5' pdaA_qPCR	tggtaaacagccatcacctataa
3' pdaA_qPCR	tccactttcatatccagcatca
5'spoIVA_qPCR	ggatagaacaagagatgagataccc
3'spoIVA_qPCR	ctgctgccttttcaaatgtc
5' spoIVB_qPCR_1	ttcagagctaggtataagtggtaat
3' spoIVB_qPCR_1	tgcggtcttccaacttctatt
5' spoIVB2_qPCR_1	agctcaaactggtggtattgt
3' spoIVB2_qPCR_1	catgtgacactgctccgatta
5' spoIIP_qPCR_1	catactcatggatgtgagacttattcaa
3' spoIIP_qPCR_1	accccatcctttgctatctaaagc
5' dpaA_qPCR	actgttattgggggagacctgc
3' dpaA_qPCR	ggaacttttggatagtgcttcagc

5' spoVAC_qPCR	agctggagctggttctataattcc
3' spoVAC_qPCR	catagccttctcttttatactccattgc
5' spoVAD_qPCR	tgacagctcagtggacagttacag
3' spoVAD_qPCR	tttggaccgtctccattagga
5' spoVAE_qPCR	gtttaatagcccaagtaatgatggatt
3' spoVAE_qPCR	acaccagttgttacatacgttaccataa
3' luciferase_ssrA_pHN149	aagcttgcatgtctgcaggcctcgagtcatcatgcagcaagtgcataattttcatcattagctgctagaatt tcttcaaaaagtctat
3' luciferase_pHN149	gccaagcttgcatgtctgcaggcctcgagtcatcatagaatttcttcaaaaag
3' PsspA_BS49	gttgttgctgttacctgagttattgttagccatgttgattaccttccttctgt
5' sspA_BS49	acacaaaataaattaaacagaaggaaggtaatcaacatggctaacaataactcagg
3' sspA_BS49	ccagtgccaagcttgcatgtctgcaggcctcgagttagaattgtcctccgcc
3' spoIVB2 F36F	attttgtgcataaattaaattatttgagaagaaatataataaaaataaaaatgttaaaac
5' spoIVB2 F36F	tacaattgttttaacatttttatttttattatatttcttctcaaataatttaatttatgc
3' spoIVB2 F37.UUA	aattttgtgcataaattaaattatttgataaaaaatataataaaaataaaaatgttaaa
5' spoIVB2 F37.UUA	caattgttttaacatttttatttttattatattttttatcaaataatttaatttatgcac
3' spoIVB2 F37.UUG	aattttgtgcataaattaaattatttgacaaaaaatataataaaaataaaaatgttaaa
5' spoIVB2 F37.UUG	caattgttttaacatttttatttttattatattttttgtcaaataatttaatttatgcac
5'sacB_UP	ttatcaggaaacagctatgaccgcggccgcgtcgactagttctttaggcccg
sacB_3'_XhoI	aagcttgcatgtctgcaggcctcgagttatttgttaactgttaattgtccttgttcaagg
3' PsspA_spoIVB2	ttaaagtattatttttaaaatgaaaatttttaagttgcatgttgattaccttccttctg
5' spoIVB2_PsspA	acaaaataaattaaacagaaggaaggtaatcaacatgcaacttaaaaattttcatt
5' spoIVB.pHN149	acaattttttatcaggaaacagctatgaccgcggccgctttattgtcttcgaatatac
3' PspoIVB_spoIVB2	aagtattatttttaaaatgaaaatttttaagttgcatatatccatctactcctatgc
5' spoIVB2_PspoIVB	aatacataataatacagcataggagtagatggatatatgcaacttaaaaattttcat
5' PspoIVB2_pHN149	attttttatcaggaaacagctatgaccgcggccgctatttatttttatgaaaactaagg
3' PspoIVB(100)_PspoIVB2	cttaataagtgatttttaatacataatatgaaaacacctctttcttatta
5' PspoIVB2_PspoIVB	ctattattaaaataatttaataagaaagaggtgttttcatattatgtattaaaaatcact

3’ PsspA_PspoIVB2	ttaattcaggtaatttttagcaattaaaacctgaaaacacctctttcttatta
5’ PsspA_PspoIVB2	ttattaaaataatttaataagaaagaggtgttttcaggttttaattgctaaaaa
3’ spoIVB2_homol	tatttttaaaatgaaaatttttaagttgcataaaacacctctttcttattaaattat
5’ spoIVB2_gene_homol	ttaaaataatttaataagaaagaggtgttttatgcaacttaaaaattttcattttaa
3’ spoIVB2end_lrgBit	tcccaatcacccacaaaatcttcaagtgtaaacaccaacatccatttaataaatacacc
5’ lrgBit_spoIVB2end	ctgtaggttatggtgtatttattaaatggatgttggtgtttacacttgaagattttgtgg
3’ spoIVB2_bitLuc	ccaatcacccacaaaatcttcaagtgtaaacaccataaaacacctctttcttattaaatt
5’ bitLuc_PspoIVB2	attattaaaataatttaataagaaagaggtgttttatggtgtttacacttgaagattttg
5’ spoIVB2_theo	ttttttatcaggaaacagctatgaccgcggccgcgatttgtcttcatttttatcttta
3’ spoIVB2_theo	gccagtgccaagcttgcatgtctgcaggcctcgagaaatatcaaagttattaatttgac

CRISPR targeting sequence is in **bold.**

Supplement Table 2. Strains and plasmids used in this study.

Strain	Description	Reference
E. coli DH5a	Cloning strain	[1]
E. coli HB101 pRK24	Conjugal donor strain, Amp^R	[2]
E. coli MB3436	*recA*$^+$ *E. coli* strain	Gift from Dr. Michael Benedik
B. subtilis BS49	*Tn*916 donor strain, Tet^R	[3]
C. difficile R20291	Wild type, ribotype 027	[4]
C. difficile CD630Δ*erm*	Wild type, ribotype 012	Gift from Dr. Daniel Paredes-Sabja (Texas A&M University)
C. difficile KNM10	R20291 *spo0A* CRISPR-Cas9 mutant	[5]
C. difficile HNN03	R20291 *sspA* CRISPR-Cas9 mutant	[6]
C. difficile HNN04	R20291 *sspB* CRISPR-Cas9 mutant with an *sspA*$_{G52V}$ allele (called *sspB** throughout this manuscript)	[6]
C. difficile HNN05	R20291 *sspA* and *sspB* CRISPR-Cas9 double mutant	[6]
C. difficile HNN17	R20291 *sspB* CRISPR-Cas9 mutant	[6]
C. difficile HNN19	EMS isolate from treatment of HNN04	This study

C. difficile HNN22	EMS isolate from treatment of HNN04	This study
C. difficile HNN26	EMS isolate from treatment of HNN04	This study
C. difficile HNN28	EMS isolate from treatment of HNN04	This study
C. difficile HNN32	EMS isolate from treatment of HNN05	This study
C. difficile HNN33	EMS isolate from treatment of HNN05	This study
C. difficile HNN35	EMS isolate from treatment of HNN05	This study
C. difficile HNN37	EMS isolate from treatment of HNN05	This study
C. difficile HNN38	EMS isolate from treatment of HNN05	This study
C. difficile HNN39	EMS isolate from treatment of HNN05	This study
C. difficile HNN40	EMS isolate from treatment of HNN05	This study
C. difficile HNN41	EMS isolate from treatment of HNN05	This study
C. difficile HNN43	CD630Δ*erm sspB* CRISPR-Cas9 mutant	This study
C. difficile HNN45	CD630Δ*erm sspA* CRISPR-Cas9 mutant	This study
C. difficile HNN46	CD630Δ*erm sspA* and *sspB* CRISPR-Cas9 double mutant	This study
C. difficile HNN48	EMS isolate from treatment of HNN05	This study
C. difficile HNN49	R20291_*0714* (*spoIVB2*) CRISPR-Cas9 mutant	This study
C. difficile HNN51	EMS isolate from treatment of HNN05	This study
C. difficile HNN57	R20291 *spoIVB2*$_{F37F}$	This study
C. difficile HNN60	R20291 *spoIVB2*$_{A20T}$	This study
C. difficile HNN64	R20291 Δ*sspA* Δ*sspB spoIVB2*$_{A20T}$	This study
C. difficile HNN73	R20291 Δ*sspA* Δ*sspB spoIVB2*$_{F37F}$	This study
**Plasmid**	**Description**	**Reference**
pMTL84151	*E. coli* – *C. difficile* shuttle vector	[7]
pMTLYN4	*traJ* containing plasmid	[8]
pJS116	*B. subtilis* – *C. difficile* shuttle vector	[9]
pKM197	CRISPR plasmid with *xylR* promoter driving *cas9*	[10]
pMB81	BitLuc containing plasmid	[12]

pJB09	*cas*9 containing plasmid for the 2-plasmid CRISPR system	[13]
pJB14	Targeting plasmid for the 2-plasmid CRISPR system	[13]
pJB94	Theophylline allelic exchange base plasmid	[14]
pJB96	pHN149 with *sacB* between *Not*I and *Xho*I cut sites, for easy selection of inserts	This study
pHN14	R20291 *sspB* promoter region and gene	[6]
pHN30	R20291 *sspA* and *sspB* complement	[6]
pHN120	CD630Δ*erm sspA* targeted CRISPR vector, gRNA 1398	This study
pHN121	CD630Δ*erm sspB* targeted CRISPR vector, gRNA 1186	This study
pHN122	CDR20291_0714 promoter region and F37F allele	This study
pHN123	CDR20291_0714 promoter region and A20T allele	This study
pHN127	CDR20291_0714 promoter region and WT allele	This study
pHN131	CD630Δ*erm sspA* targeted CRISPR vector with *TraJ oriT,* gRNA 165	This study
pHN132	CD630Δ*erm sspB* targeted CRISPR vector with *TraJ oriT*, gRNA 144	This study
pHN138	CD630Δ*erm sspA* targeted CRISPR vector with *TraJ oriT*, gRNA 135	This study
pHN145	CDR20291_0714 promoter region and S301A allele	This study
pHN146	CDR20291_0714 promoter region and F37F, S301A allele	This study
pHN147	CDR20291_0714 promoter region and A20T, S301A allele	This study
pHN149	pMTL84151 based plasmid that also contains the *Tn916 oriT* (base plasmid that can be conjugated through *E. coli* or *B. subtilis* conjugal donors)	This study
pHN152	CD630Δ*erm sspA* promoter region and gene	This study
pHN153	CD630Δ*erm sspA* and *sspB* promoter region and gene	This study
pHN157	CDR20291_0714 targeted CRISPR vector, gRNA 3	This study
pHN176	CD630Δ*erm sspB* promoter region and gene	This study
pHN208	CDR20291_0714 promoter region and F36F	This study
pHN218	CDR20291_0714 promoter region and F37L (UUA codon)	This study
pHN219	CDR20291_0714 promoter region and F37L (UUG codon)	This study
pHN220	*sspA* promoter region and *sspA* gene from *B. subtilis* BS49	This study
pHN271	*spoIVB2*A20T theophylline allelic exchange	This study

pHN272	*spoIVB2*F37F theophylline allelic exchange	This study
pHN312	*sspA* promoter driving *spoIVB2* expression	This study
pHN329	*spoIVB* promoter driving *spoIVB2* expression	This study
pHN330	*spoIVB2* and *spoIVB* promoters driving *spoIVB2* expression	This study
pHN331	*spoIVB2* and *sspA* promoters driving *spoIVB2* expression	This study
pHN335	*spoIVB2* promoter with *spoIVB2* attached to *bitLuc* (luciferase) and tagged with *ssrA*	This study
pHN336	*spoIVB2* promoter with *spoIVB2*$_{A20T}$ attached to *bitLuc* (luciferase) and tagged with *ssrA*	This study
pHN337	*spoIVB2* promoter with *spoIVB2*$_{F37F}$ attached to *bitLuc* (luciferase) and tagged with *ssrA*	This study
pHN338	*spoIVB2* promoter with *bitLuc* (luciferase) and tagged with *ssrA*	This study
pHN339	*spoIVB2* promoter with *bitLuc* (luciferase)	This study

1. Hanahan D. Studies on transformation of *Escherichia coli* with plasmids. J Mol Biol. 1983;166(4):557-80. Epub 1983/06/05. doi: 10.1016/s0022-2836(83)80284-8. PubMed PMID: 6345791.
2. Ma NJ, Moonan DW, Isaacs FJ. Precise manipulation of bacterial chromosomes by conjugative assembly genome engineering. Nature protocols. 2014;9(10):2285-300. doi: 10.1038/nprot.2014.081.
3. Bouillaut L, McBride SM, Sorg JA. Genetic Manipulation of *Clostridium difficile*. Current Protocols in Microbiology2011.
4. Stabler RA, He M, Dawson L, Martin M, Valiente E, Corton C, et al. Comparative genome and phenotypic analysis of *Clostridium difficile* 027 strains provides insight into the evolution of a hypervirulent bacterium. Genome Biol. 2009;10(9):R102. Epub 2009/09/29. doi: 10.1186/gb-2009-10-9-r102. PubMed PMID: 19781061; PubMed Central PMCID: PMCPMC2768977.
5. McAllister KN, Martinez Aguirre A, Sorg JA. The selenophosphate synthetase, *selD*, is important for *Clostridioides difficile* physiology. BioRxIV. 2021. doi: 10.1101/2021.01.06.425661.
6. Nerber HN, Sorg JA. The small acid-soluble proteins of *Clostridioides difficile* are important for UV resistance and serve as a check point for sporulation. PLoS Pathog. 2021;17(9):e1009516. Epub 2021/09/09. doi: 10.1371/journal.ppat.1009516. PubMed PMID: 34496003.
7. Heap JT, Pennington OJ, Cartman ST, Minton NP. A modular system for *Clostridium* shuttle plasmids. J Microbiol Methods. 2009;78(1):79-85. Epub 20090513. doi: 10.1016/j.mimet.2009.05.004. PubMed PMID: 19445976.
8. Ng YK, Ehsaan M, Philip S, Collery MM, Janoir C, Collignon A, et al. Expanding the repertoire of gene tools for precise manipulation of the *Clostridium difficile* genome: allelic exchange using *pyrE* alleles. PLoS One. 2013;8(2):e56051. Epub 2013/02/14. doi: 10.1371/journal.pone.0056051. PubMed PMID: 23405251; PubMed Central PMCID: PMCPMC3566075.

9. Sorg JA, Sonenshein AL. Bile salts and glycine as cogerminants for *Clostridium difficile* spores. J Bacteriol. 2008;190(7):2505-12. Epub 2008/02/05. doi: 10.1128/JB.01765-07. PubMed PMID: 18245298; PubMed Central PMCID: PMCPMC2293200.

10. Bhattacharjee D, Sorg JA. Factors and Conditions That Impact Electroporation of *Clostridioides difficile* Strains. mSphere. 2020;5(2). Epub 2020/03/07. doi: 10.1128/mSphere.00941-19. PubMed PMID: 32132157; PubMed Central PMCID: PMCPMC7056809.

12. Baloh MaS, J.A. . *Clostridioides difficile* SpoVAD and SpoVAE Interact and Are Required for Dipicolinic Acid Uptake into Spores. J Bacteriol. 2021;203(21). doi: https://doi.org/10 .1128/JB.00394-21.

13. Brehm JN, Sorg JA. Plasmid Sequence and Availability for an Improved *Clostridioides difficile* CRISPR-Cas9 Mutagenesis System. Microbiol Resour Announc. 2022;11(12):e0083322. Epub 20221107. doi: 10.1128/mra.00833-22. PubMed PMID: 36342279; PubMed Central PMCID: PMCPMC9753633.

14. Brehm JN, Sorg JA. Plasmid sequence and availability for an improved *Clostridioides difficile* CRISPR-Cas9 mutagenesis system Microbiol Resour Announc. 2022;11(12). doi: 10.1128/mra.00833-22.

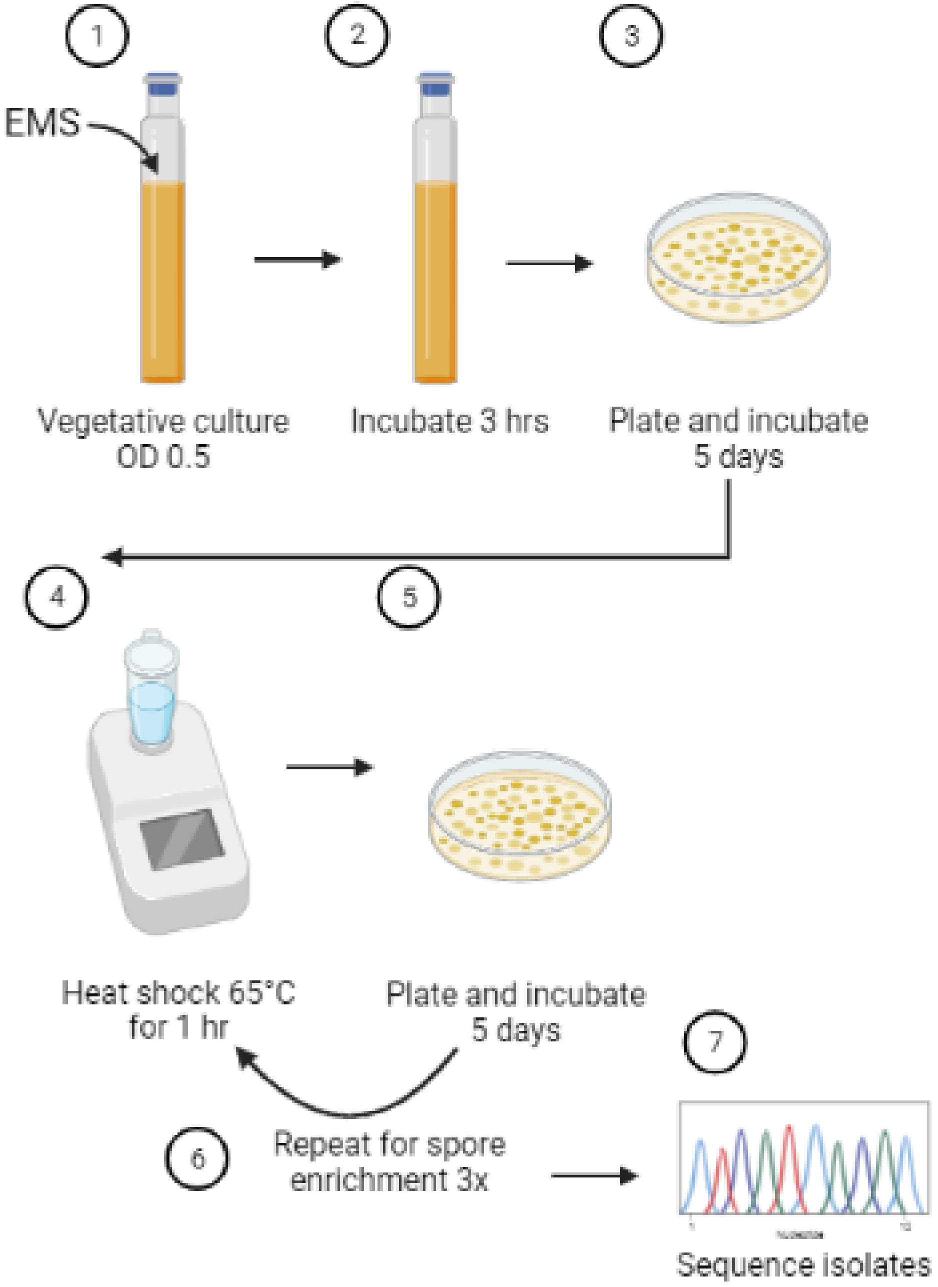
1
2
3
EMS
Vegetative culture
OD 0.5
Incubate 3 hrs
Plate and incubate
5 days
4
5
Heat shock 65°C
for 1 hr
Plate and incubate
5 days
6
Repeat for spore
enrichment 3x
7
Sequence isolates

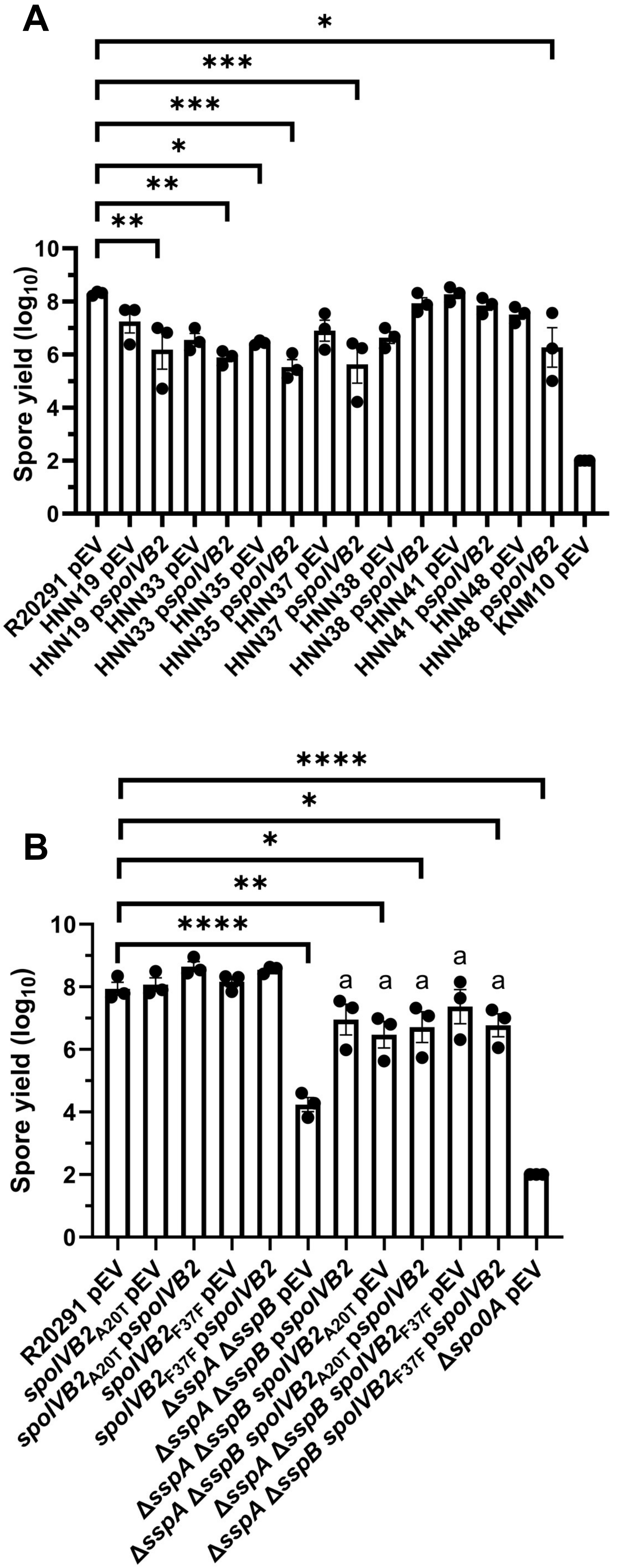
A
*
***
***
*
**
**
Spore yield ($\log_{10}$)
0
2
4
6
8
10
R20291 pEV
HNN19 pEV
HNN19 pspoIVB2
HNN33 pEV
HNN33 pspoIVB2
HNN35 pEV
HNN35 pspoIVB2
HNN37 pEV
HNN37 pspoIVB2
HNN38 pEV
HNN38 pspoIVB2
HNN41 pEV
HNN41 pspoIVB2
HNN48 pEV
HNN48 pspoIVB2
KNM10 pEV
B
****
*
*
**
****
a
a
a
a
a
Spore yield ($\log_{10}$)
0
2
4
6
8
10
R20291 pEV
spoIVB2$_{A20T}$ pEV
spoIVB2$_{A20T}$ pspoIVB2
spoIVB2$_{F37F}$ pEV
spoIVB2$_{F37F}$ pspoIVB2
ΔsspA ΔsspB pEV
ΔsspA ΔsspB pspoIVB2
ΔsspA ΔsspB spoIVB2$_{A20T}$ pEV
ΔsspA ΔsspB spoIVB2$_{A20T}$ pspoIVB2
ΔsspA ΔsspB spoIVB2$_{F37F}$ pEV
ΔsspA ΔsspB spoIVB2$_{F37F}$ pspoIVB2
Δspo0A pEV

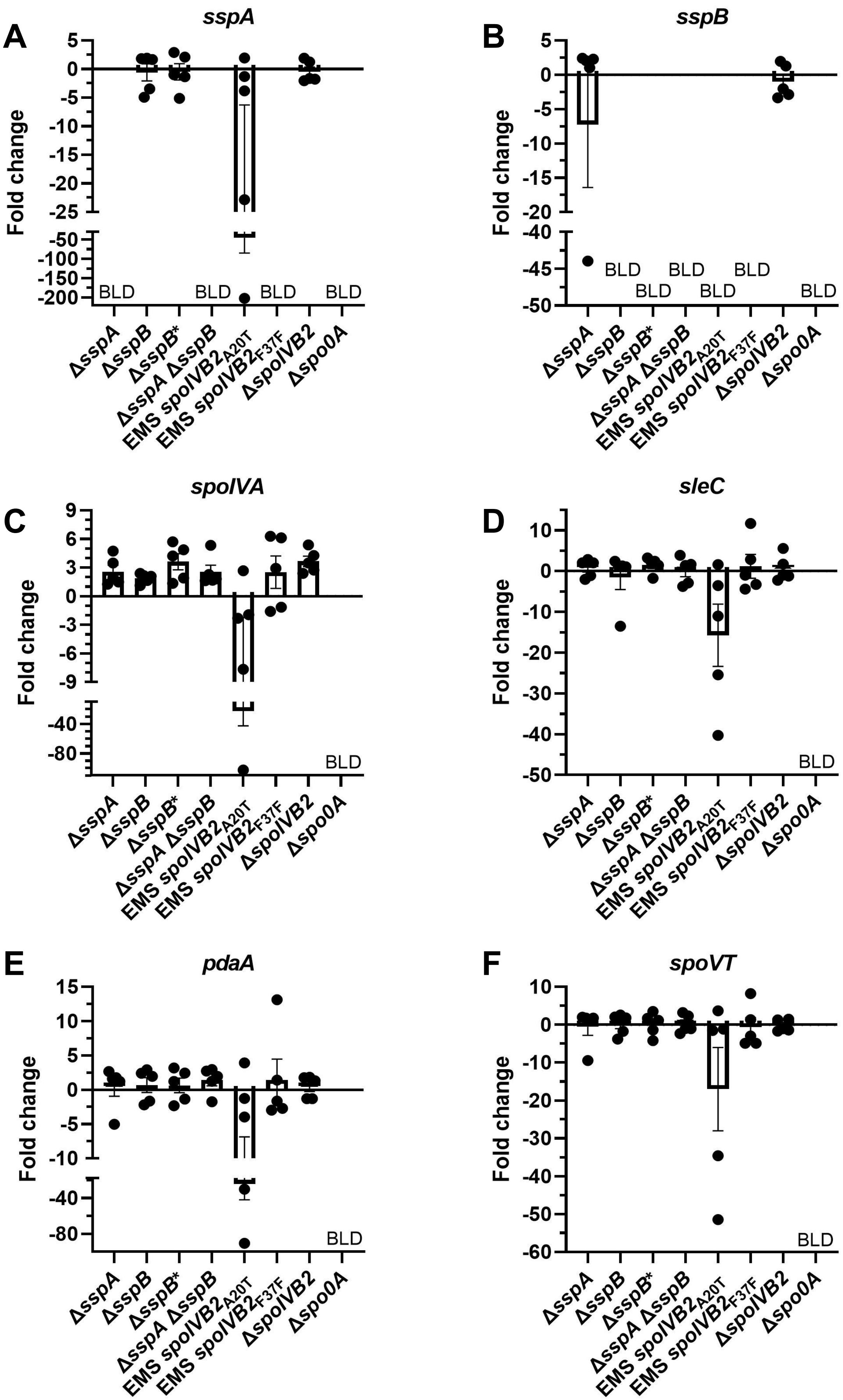
A
sspA
Fold change
BLD
BLD
BLD
BLD
B
sspB
Fold change
BLD
BLD
BLD
BLD
BLD
BLD
C
spoIVA
Fold change
BLD
D
sleC
Fold change
BLD
E
pdaA
Fold change
BLD
F
spoVT
Fold change
BLD
ΔsspA
ΔsspB
ΔsspB*
ΔsspA ΔsspB
EMS spoIVB2A20T
EMS spoIVB2F37F
ΔspoIVB2
Δspo0A

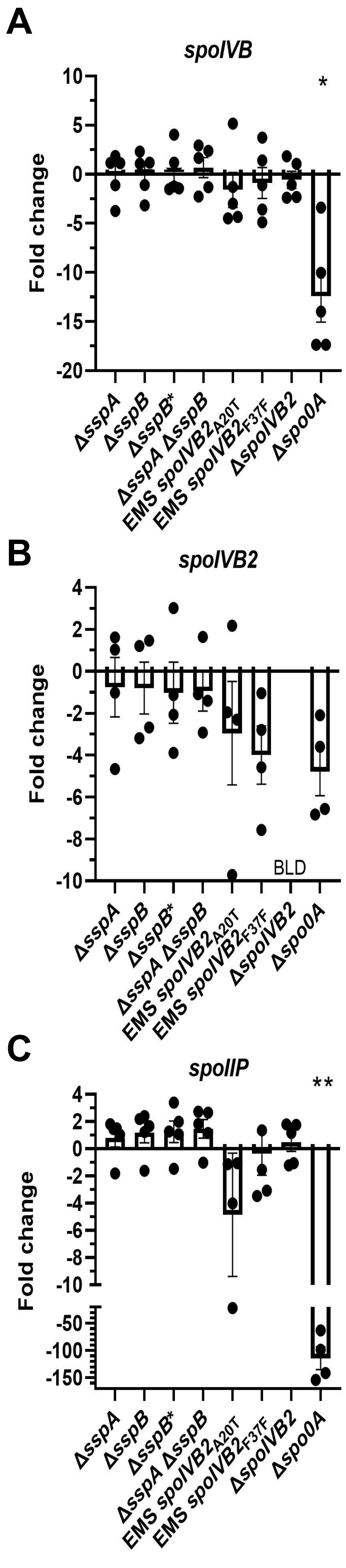
A
spoIVB
B
spoIVB2
BLD
C
spoIIP
Fold change
ΔsspA
ΔsspB
ΔsspB*
ΔsspA ΔsspB
EMS spoIVB2A20T
EMS spoIVB2F37F
ΔspoIVB2
Δspo0A

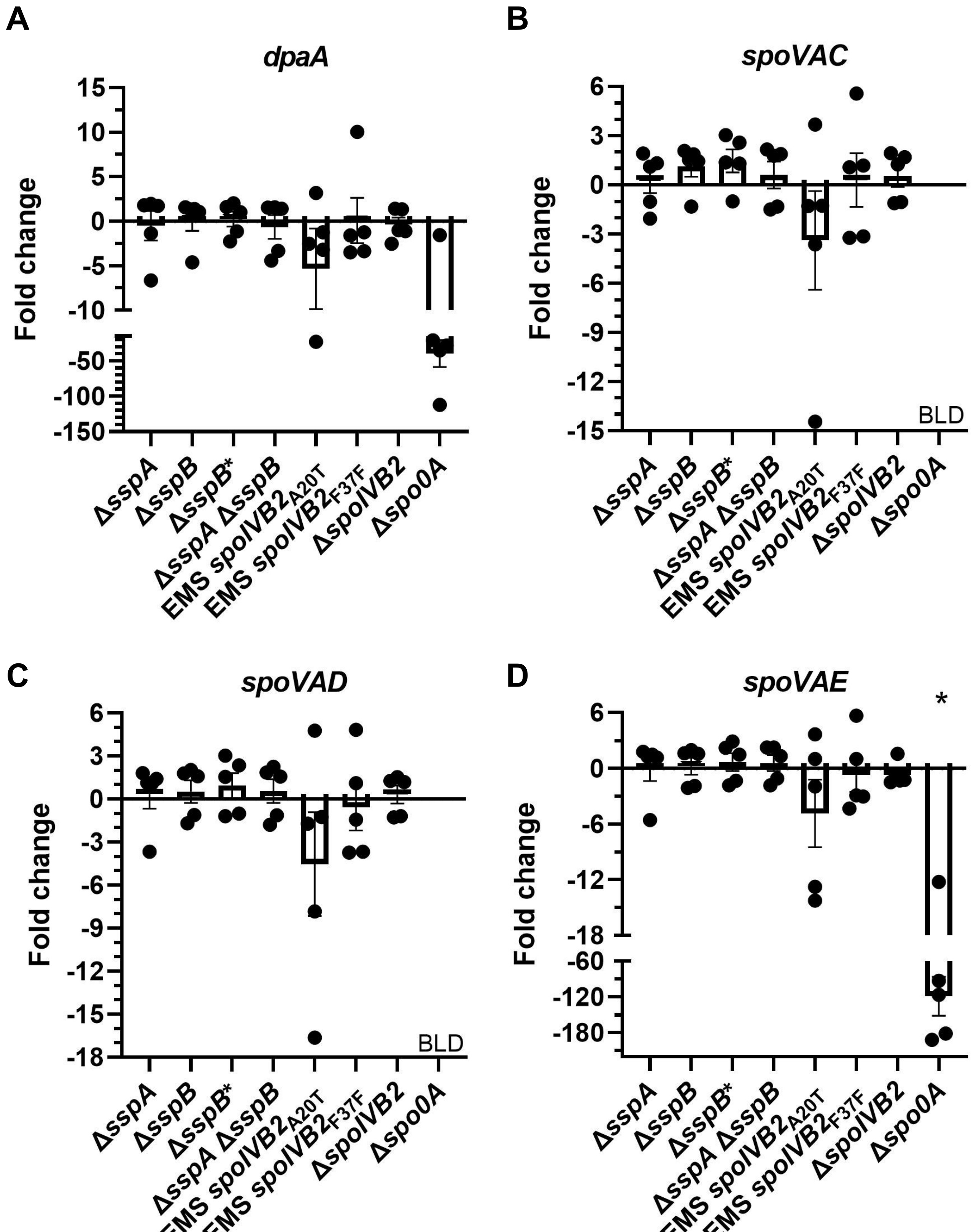
A
dpaA
Fold change
15
10
5
0
-5
-10
-50
-100
-150
B
spoVAC
6
3
0
-3
-6
-9
-12
-15
BLD
C
spoVAD
-15
-18
D
spoVAE
*
-18
-60
-120
-180
ΔsspA
ΔsspB
ΔsspB*
ΔsspA ΔsspB
EMS spoIVB2A20T
EMS spoIVB2F37F
ΔspoIVB2
Δspo0A
